# Spatially Resolved Near Field Spectroscopy of Vibrational Polaritons at the Small N Limit

**DOI:** 10.1021/acsphotonics.4c00345

**Published:** 2024-06-20

**Authors:** Oliver Hirschmann, Harsh H. Bhakta, Wilton J. M. Kort-Kamp, Andrew C. Jones, Wei Xiong

**Affiliations:** † Department of Chemistry and Biochemistry, University of California San Diego, La Jolla, California 92093, United States; ‡ Theoretical Division, Los Alamos National Laboratory, Los Alamos, New Mexico 87545, United States; § Center for Integrated Nanotechnologies, Materials Physics and Applications Division, Los Alamos National Laboratory, Los Alamos, New Mexico 87545, United States; ∥ Materials Science and Engineering Program, University of California San Diego, La Jolla, California 92093, United States; ⊥ Department of Electrical and Computer Engineering, University of California San Diego, La Jolla, California 92093, United States

**Keywords:** molecular vibrational polariton, s-SNOM, strong coupling, quartz micropillar, surface phonon polariton

## Abstract

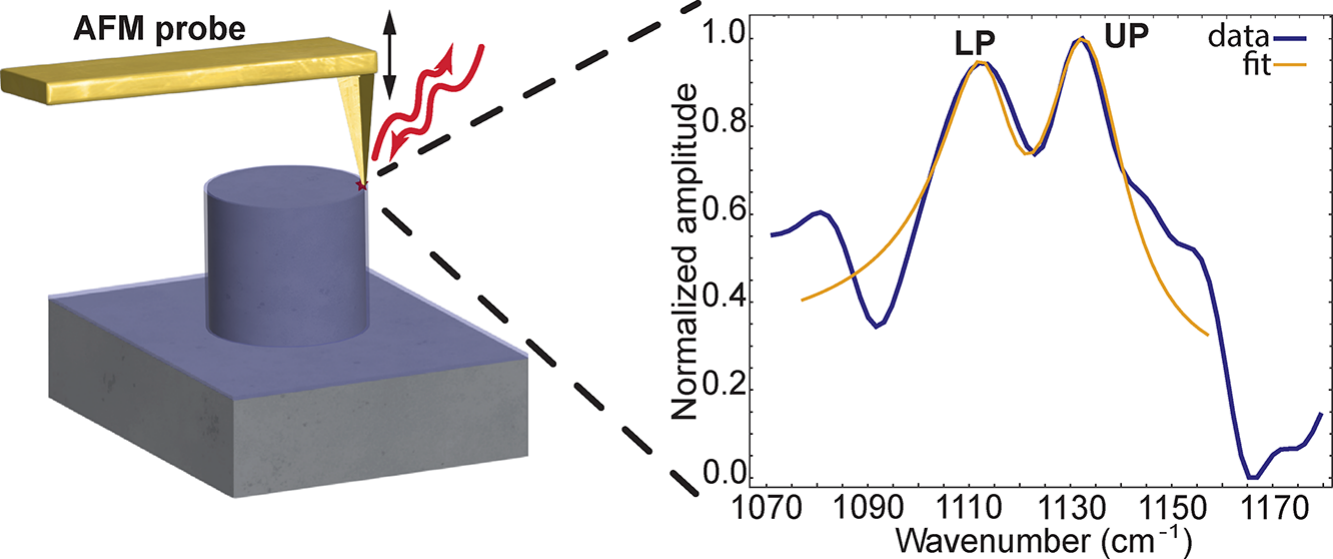

Vibrational polaritons, which have been primarily studied in Fabry–Pérot cavities with a large number of molecules (*N* ∼ 10^6^–10^10^) coupled to the resonator mode, exhibit various experimentally observed effects on chemical reactions. However, the exact mechanism is elusively understood from the theoretical side, as the large number of molecules involved in an experimental strong coupling condition cannot be represented completely in simulations. This discrepancy between theory and experiment arises from computational descriptions of polariton systems typically being limited to only a few molecules, thus failing to represent the experimental conditions adequately. To address this mismatch, we used surface phonon polariton (SPhP) resonators as an alternative platform for vibrational strong coupling. SPhPs exhibit strong electromagnetic confinement on the surface and thus allow for coupling to a small number of molecules. As a result, this platform can enhance nonlinearity and slow down relaxation to the dark modes. In this study, we fabricated a pillar-shaped quartz resonator and then coated it with a thin layer of cobalt phthalocyanine (CoPc). By employing scattering-type scanning near-field optical microscopy (s-SNOM), we spatially investigated the dependency of vibrational strong coupling on the spatially varying electromagnetic field strength and demonstrated strong coupling with 38,000 molecules only—reaching to the small N limit. Through s-SNOM analysis, we found that strong coupling was observed primarily on the edge of the quartz pillar and the apex of the s-SNOM tip, where the maximum field enhancement occurs. In contrast, a weak resonance signal and lack of coupling were observed closer to the center of the pillar. This work demonstrates the importance of spatially resolved polariton systems in nanophotonic platforms and lays a foundation to explore polariton chemistry and chemical dynamics at the small N limit—one step closer to reconcile with high-level quantum calculations.

## Introduction

Molecular vibrational strong coupling (VSC) occurs when a molecular vibration and an optical resonance mode strongly interact with each other in such a way that the energy exchange rate between the molecular vibration and cavity mode surpasses their individual dephasing lifetime.^1−4^ One observable consequence is the emergence of two distinct optical bright modes known as the lower polariton (LP) and the upper polariton (UP). When strongly coupled, they exhibit a separation, called vacuum Rabi splitting (Ω), that is greater than the individual line widths of the molecular and cavity modes.^3,5,6^ The study of molecular vibrational polaritons (MVPs), which involves the strong coupling between molecular vibrations and optical resonance modes,^5,6^ holds significant promise in diverse areas such as modifying chemical reactions,^2,7−13^ altering energy transfer pathways,^14−17^ and serving as a platform for quantum simulations.^1,18−23^

MVPs have predominantly been studied using Fabry–Pérot microcavity systems. In such systems, due to the large cavity volume (>10^5^ μm^3^), a large number of molecules (*N* ∼ 10^6^–10^10^) are required to achieve the so-called collective strong coupling.^24−26^ Consequently, aside from the two bright modes LP and UP that show dispersive characteristics in a Fabry–Pérot cavity, there exists an ensemble of *N* – 1 new eigenstates with asymmetric combinations of matter wave functions and no photon contributions, referred as dark modes.^2,3^ The nature and influence of these dark modes, which are theoretically described to be primarily molecular in origin, are subject of an ongoing debate.^27^ Experimental studies have demonstrated that changes in reaction rates can be observed under collective strong coupling in Fabry–Pérot systems even though there are ∼10^6^–10^10^ dark modes and only two bright modes (LP and UP).^11,27,28^ In another work from our group, researchers separately resolved an ultrafast isomerization dynamic rate of polaritons and dark modes and concluded that although polaritons unambiguously can promote vibrational energy distributions and suppress the isomerization rate, dark modes remain irrelevant, as expected because of their primarily molecular characters.^10^ This research, however, pointed out a viable general direction for enhancing polariton modified chemistry—that is, reducing the number of dark modes by enhancing the vacuum electromagnetic field (*E*) and reducing the photonic mode volume (*V*).

Indeed, while experiments are conducted in the collective strong coupling regime, theoretical studies that reported polariton-modified chemistry were typically limited to only a few molecules and dark modes, exacerbating the mismatch between theory and experiment. This gap hampers our understanding of whether the coupling strength is distributed among a large *N* number of molecules and how it can influence molecular properties, now often referred to as the big *N* problem.^29^ Thus, to reduce the gap between the experimental and theoretical works of polariton chemistry, and to enhance our understanding of the role played by dark modes, it becomes essential to bridge them and develop experimental systems that facilitate collective strong coupling with a reduced number of molecules, while theorists work on methods to expand the number of simulated molecules, which started to emerge recently.^30^ Currently, the *N* needs to be small enough so that high-level theory can handle the size of the simulation,^31−34^ while experimentalists can also generate polaritons and study their influence on chemistry—allowing both sides to advance our understanding of the energy redistribution of MVPs. While an ideal scenario for studying vibrational strong coupling involves a system with a single molecule that avoids dark modes completely, achieving this level is formidable and so far, only being reported with electronic transitions of selective systems that have a giant transition dipole moment,^35^ or at a cryotemperature,^36^ with the former not applicable to vibrational modes. Therefore, the current focus is to achieve a VSC with a small number of molecules. Considering that computational simulations have shown studies of collective strong coupling up to a few thousand molecules, and the polaritons still can affect chemistry,^27^ we aim to reach collective strong coupling with *N* ∼ 10^4^, referring to it as the small N limit.

Achieving this goal is challenging, but could be accomplished through stronger *E* confinement. For a strongly coupled system, the electromagnetic field depends on mode volume *V* by ,^7,37^ leading to
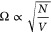
1

Thus, naturally, a confined optical mode enhances the *E* field and could enable strong coupling at the small *N* limit. In return, achieving strong coupling at the small *N* limit allows for a reduction in the number of dark modes, while the number of LP and UP remains constant. This alignment is expected to lead to enhanced nonlinear effects, and to reduce energy redistribution channels and rates through dark modes.^38^

One promising approach involves materials that exhibit a Reststrahlen band and support resonators that are based on a surface phonon polaritons (SPhPs) mode, offering excellent confinement within small mode volumes.^39−41^ SPhPs are quasiparticles resulting from the coupling between photons and optical phonons.^42^ SPhPs have been observed in the Reststrahlen band of crystals, with this band featuring a negative real part of the conductivity.^43,44^ This allows for deeply subdiffractional mode confinement and therefore demonstrates lower loss rates compared to their plasmonic counterparts.^45^ Leveraging the inverse square root relationship between Ω and *V*, SPhPs provide an ideal platform for achieving the VSC at the small *N* limit. Hillenbrand was the first to demonstrate the small *N* limit strong coupling between a vibrational mode and a SPhP resonator using h-BN ribbons.^45,46^ Subsequently, Wang expanded on this concept by employing a quartz resonator and successfully achieved coupling with an organic molecule −4-nitrobenzyl alcohol.^44^

**Figure 1:**
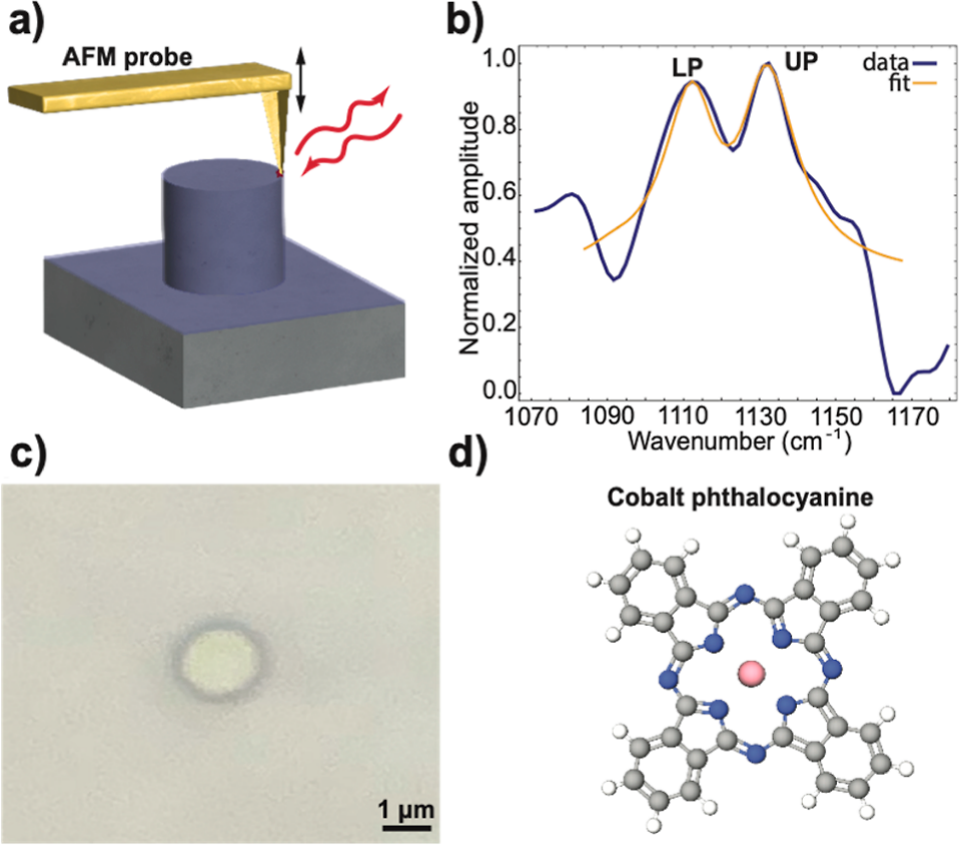
Tip-enhanced near field measurement of a quartz pillar covered with a thin layer of cobalt phthalocyanine. (a) A schematic drawing of a s-SNOM measurement on the resonator done by the scattering of light from a sharp AFM probe on the edge of the pillar. (b) s-SNOM obtained spectrum (blue) from the edge of the 20 nm thick CoPc covered pillar, showing signatures of lower and upper polariton (LP and UP). The yellow line shows the corresponding fit. (c) A micrograph of a 1.6 μm in diameter and 1.3 μm tall quartz pillar fabricated by reactive ion etching (RIE). (d) Cobalt phthalocyanine model. Pink: cobalt, gray: carbon, blue: nitrogen, and white: hydrogen.

Building upon their work, this study investigated the quartz resonator further by demonstrating VSC with a different molecule—cobalt phthalocyanine (CoPc) and using scattering-scanning near-field optical microscopy (s-SNOM) techniques with 20 nm spatial resolution (Figure 1a)^47^ (see Supporting Information, Section S6) to explore the spatial dependence of the coupling strength. We revealed that the coupling (Figure 1b) is confined primarily to the edge of the pillar and the apex of the s-SNOM tip, where the electromagnetic field is most confined and enhanced. We found that different from Wang’s report, where a perfect nanophotonic pillar was used in the simulation, a pillar with a rounded edge agrees with our experimental results better. The rounded edge leads to reduced field enhancement with longer field penetration, which together resulted in multiple molecular layers necessary for reaching strong coupling, in contrast to the monolayer concluded before.^44^ Through this approach, we determined that ∼38,000 molecules participate in the VSC under the probing volume. The ability to precisely track a reduced number of molecules through a combined experimental and theoretical approach, by taking into account the imperfection of the nanofabrication process, is instrumental in obtaining realistic estimation of the total number of molecules in nanophotonic environments for further enhancing nonlinearity and coherence time.

## Results and Discussion

To achieve VSC, we fabricated single quartz pillars with diameters ranging from 1 to 2.5 μm and a height of 1.3 μm (Figure 1c, Supporting Information, Section S1). These pillars were etched from a quartz crystal (see the Methods). Following fabrication, the surface of the pillars was uniformly coated with CoPc (Figure 1d) using an Organic Knudsen cell in ultrahigh vacuum (setup shown in Supporting Information, Section S1). CoPc was chosen due to its sharp absorption peak at approximately 1120 cm^–1^ (see Supporting Information, Section S5), which falls within the Reststrahlen band region (where Re[ε_quartz_] < 0) of the α-quartz crystal.^44^ This peak at 1120 cm^–1^ is originated from an in-plane deformation of C–H.^48^ In addition, CoPc is widely used in chemistry, such as an electrocatalyst for CO_2_^49^ and in organic solar cells.^50^ Thus, reaching the VSC could potentially provide a new pathway to control its photochemical properties and reactivities.

**Figure 2:**
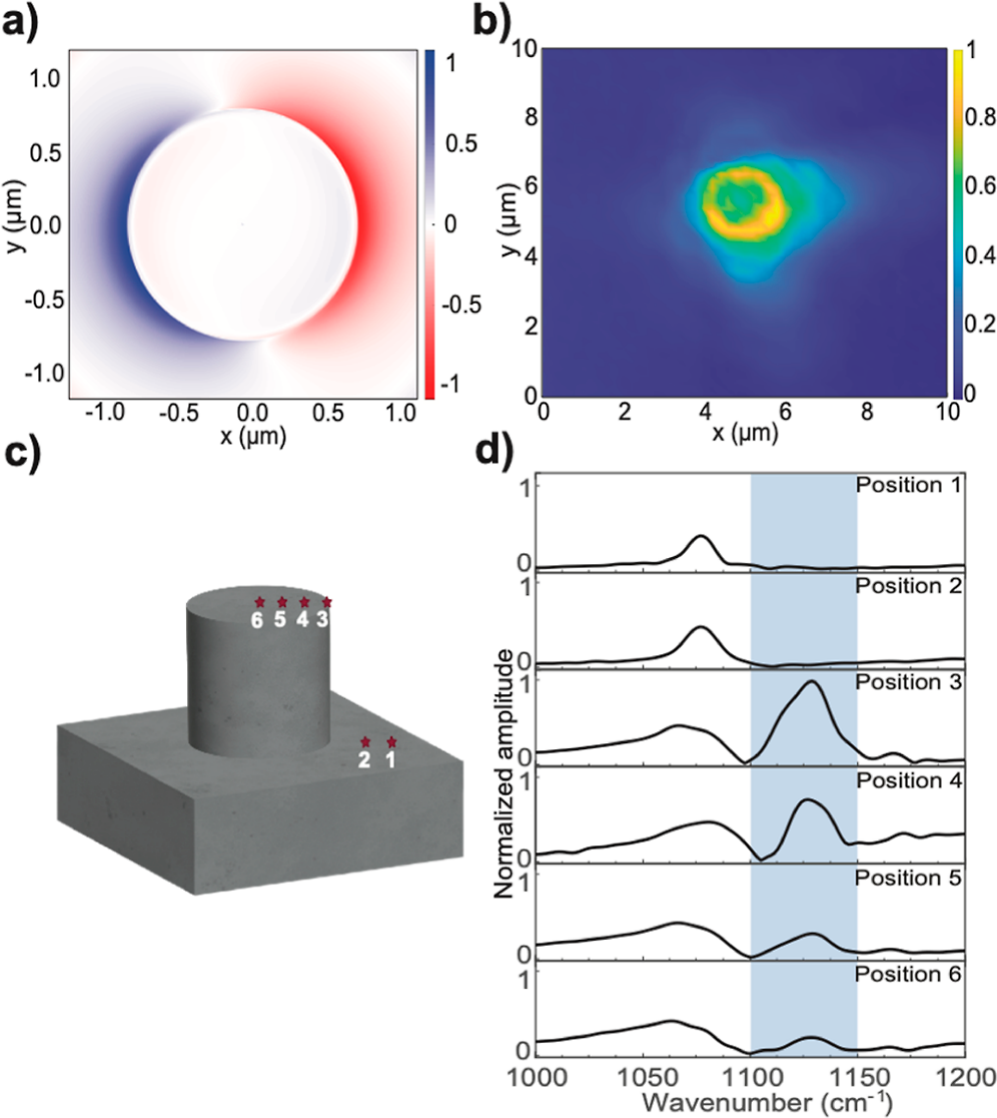
Field confinement of a 1.6 μm diameter and 1.3 μm tall quartz pillar resolved by OPTIR and s-SNOM. (a) A FDTD simulation of the pillar shows the confined cavity mode’s *E*_*x*_ field distribution at the top edge of the pillar. The corresponding side view profile is shown in Supporting Information, Figure S6. (b) Spatially resolved enhancement of the electromagnetic field measured by OPTIR at 1129 cm^–1^. Yellow indicates the maximum intensity, hence field enhancement, and blue shows no intensity measured, hence no field enhancement. (c) Marked positions and (d) the corresponding IR spectra of a quartz pillar obtained with s-SNOM. A small peak around 1080 cm^–1^ is observed outside the pillar and a strong peak at 1129 cm^–1^ is detected at the edge, with decreasing amplitude toward the center. All spectra are normalized with respect to the maximum intensity of the position 3–peak in the blue range at ∼1129 cm^–1^ (see Supporting Information, Section S7).

To understand the SPhP resonance, we performed finite-difference time-domain (FDTD) simulations of the quartz pillars using the MIT electromagnetic equation propagation (MEEP) software.^51^ The simulations predicted significant field confinement at the edge of the quartz pillar (Figure 2a, Supporting Information, Section S2). To validate the simulations, we conducted spatial imaging of a 1.6 μm diameter pillar using optical photothermal infrared (OPTIR) spectroscopy^52−54^ at 1129 cm^–1^ (Figure 2b). As anticipated from the simulation, we observed enhanced signal intensity around the edge of the pillar (highlighted in yellow). It is important to note that due to thermal effects detected by OPTIR, the measured enhancement extends slightly beyond the simulated field confinement.

To further characterize the spatial distribution of the SPhP mode, we employed s-SNOM^47,55,56^ measurements on a similar-sized pillar. Outside the pillar, a weak peak at approximately 1080 cm^–1^ was observed from the intrinsic quartz resonance (Figure 2c,d, positions 1 and 2). The maximum enhancement of the electromagnetic field due to pillar confinement, in accordance with the simulation predictions, was found exclusively at the edge of the pillar (position 3). As the measurement was conducted closer to the center of the pillar (positions 4–6), the observed enhancement became weaker. This spatial dependence of the electromagnetic field strength aligns well with the simulation results. On top of the pillar (positions 3–6), we observed a broadening of the intrinsic quartz peak. We propose that this line shape change could be due to a less confined mode of the pillar interacting with the intrinsic quartz peak, which is out of the focus of this work.

**Figure 3:**
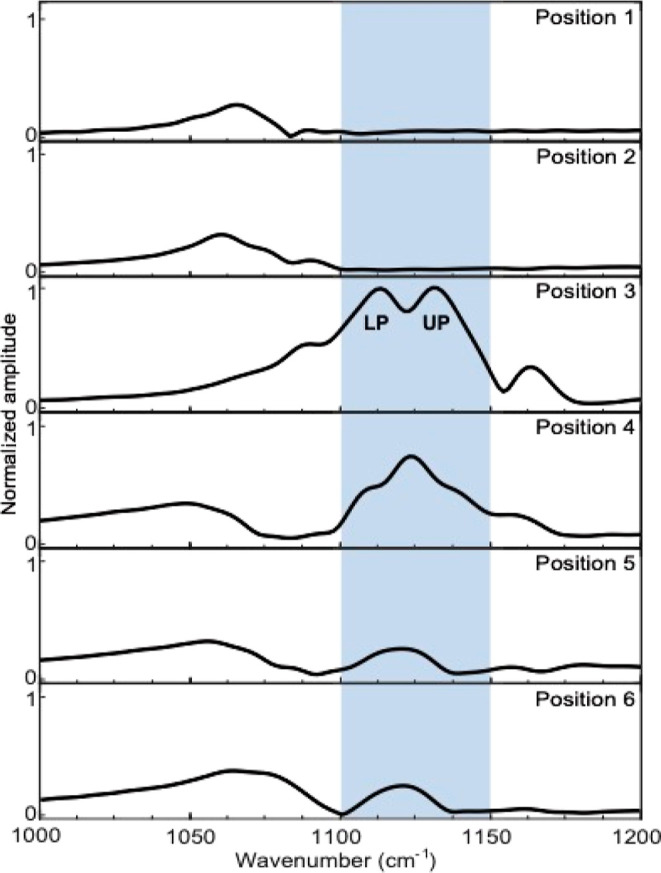
Field confinement and Rabi splitting on the edge of a quartz pillar like Figure 2 but covered with Cobalt phthalocyanine is shown, measured with s-SNOM. Outside the pillar (position 1 and 2) a weak peak around 1070 cm^–1^ can be seen, while position 3 at the edge of the pillar shows clear Rabi splitting signature and positions 4–6 show a decreasing resonator peak intensity with no noticeable Rabi splitting signature. Similar to Figure 2, all spectra are normalized with respect to the maximum intensity of position 3–blue range (see Supporting Information, Section S6).

After depositing the CoPc layer, we repeated the s-SNOM measurement and observed a slightly shifted peak at approximately 1070 cm^–1^ outside the pillar (Figure 3, positions 1 and 2), due to a change of refractive indices. At position 3, corresponding to the edge of the pillar, a significant Rabi splitting of 20 cm^–1^ was observed. We note that s-SNOM measures amplitude, which resembles absorption in the case of strong coupling.^57^ Moving toward position 4, the spectrum exhibited a shifted resonator peak at 1121 cm^–1^, along with additional features below and above this peak, which are believed to be related to Rabi splitting. As we moved further towards the center of the pillar (positions 5 and 6), there was a noticeable decrease in the intensity of the resonator peak at 1121 cm^–1^, and no clear signature of Rabi splitting was observed. These results are in line with the E-field distribution and corroborate the findings reported by Wang and colleagues.^44^ Importantly, in this study, we spatially resolved the field enhancement and demonstrated that the Rabi splitting is primarily confined to the edge of the pillar, confirming the statement previously concluded based on a simulation effort only.^44^ This observation, corroborated by the agreement between our simulations and experimental measurements, reinforces the robustness and validity of our findings: The spatial confinement of VSC to the edge of the pillar emphasizes its crucial role in driving the VSC phenomenon, while other areas of the pillars exhibit limited light-matter coupling.

To further characterize the polariton features observed in Figure 3, position 3, we conducted detuning experiments using s-SNOM, by varying the diameter of the pillars between 1 and 2.5 μm. Because the resonant frequency of the SPhPs is primarily determined by the diameter of the pillar, this allows us to tune its frequency within the Reststrahlen band. Importantly, all the pillars with different diameters were fabricated on the same quartz crystal to ensure a consistent molecular layer thickness among pillars, as only one deposition is necessary. To comprehensively characterize the dispersion curve as a function of coupling strength, the same detuning experiments were conducted on pillars with different molecular film thickness, ranging from 8, 20, 30, and 50 nm.

We then fitted the measured spectra to extract quantitative information about the detuning and coupling strength. In the fitting model, we included both the SPhP mode and the molecular vibration at ω_m_ = 1122 cm^–1^, treating them as classical harmonic oscillators coupled by the coupling strength *g*. Note that we also explored including other vibrational modes to couple to the cavity, and they manifest little influence on the spectral fitting due to their far detuned frequencies. We thereby chose a model with a minimal number of oscillators. The equation is determined by the following description.^46^
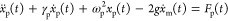
2
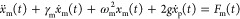
3

**Figure 4:**
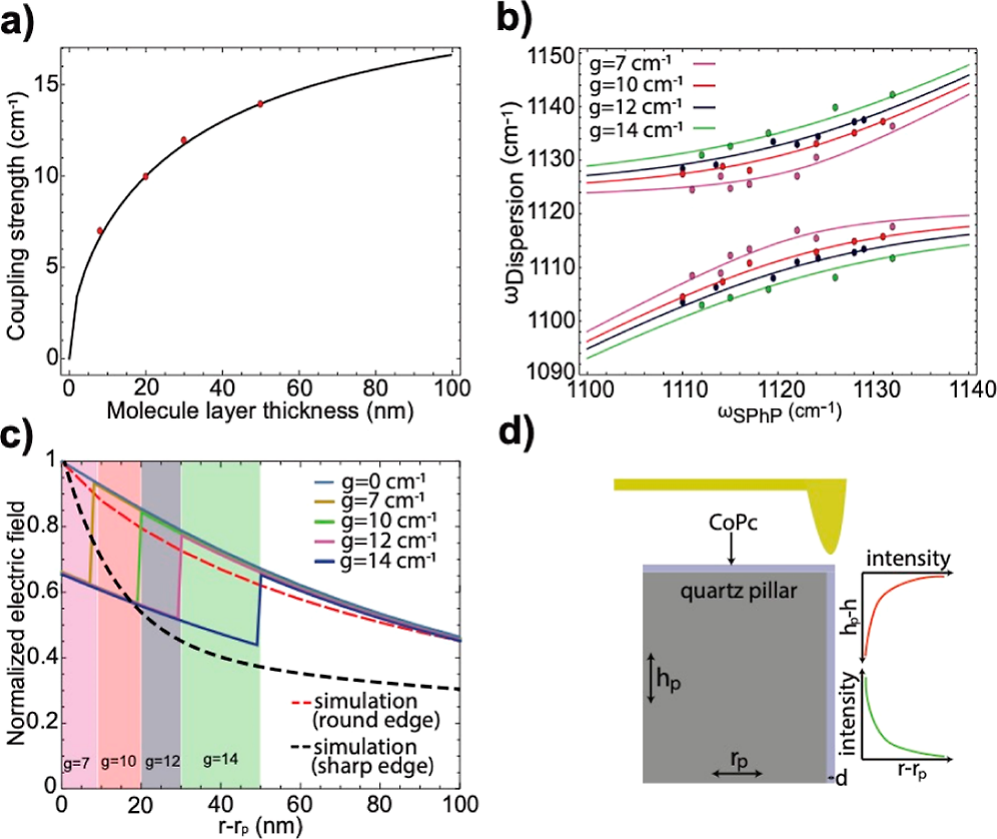
Quantitative analysis of VSC at the small N limit. (a) Distribution of coupling strength as a function of molecule layer thickness. The black line is the result of fitting eq 6 to the coupling strength (red dots), determined from eq 5. (b) The dispersion curve for different coupling strengths that result from different CoPc layer thicknesses (8 nm is *g* = 7 cm^–1^, 20 nm is *g* = 10 cm^–1^, 30 nm is *g* = 12 cm^–1^, and 50 nm is *g* = 14 cm^–1^, the pillar diameter can be found in Supporting Information, Section S7, Tables S5–S8). (c) Experimentally determined electromagnetic field distribution (lines), shown for the different coupling strengths/layer thicknesses and the FDTD simulation (red dashed curve for a rounded edge and black dashed curve for a sharp edge) obtained counterpart without molecules. The different layer thicknesses are shown by the colored region. (d) Illustration of the pillar edge measured with s-SNOM and a schematic of the electromagnetic field decay.

The harmonic oscillators in our system are characterized by their respective frequencies ω_p_, ω_m_, as well as their damping rates γ_p_, γ_m_. The SPhP mode is represented by *x*_*p*_ (*t*), while the molecular mode is described by *x*_m_ (*t*), where the dots indicate time derivatives. The coupling strength is determined by the parameter *g*, *F*_p_ (*t*) and *F*_m_ (*t*) represent the effective forces that drive the resonator. In this experiment, we set *F*_m_ (*t*) to 0 because the near field of the tip does not significantly interact with the molecules due to the weaker external illumination source compared to the strong resonator enhancement.^46^ Based on eqs 2 and 3, the solution for the detected field is eq 4, which was utilized for fitting (see Supporting Information, Section S3).
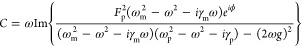
4In 4, the term *e*^*i*ϕ^ accounts for the drift of the interferometer between the measurement and reference.^46^ A representation of the fitting is depicted in Figure 1b as a yellow curve. During the fitting procedure, we considered the following parameters as free variables, γ_p_, ω_p_, γ_m_, ω_m_, *F*_p_ (*t*), *g*, and ϕ, while ensuring they remained within a physically realistic range. Based on the fitting results, we determined the eigenfrequencies of LP and UP, denoted as ω_–_ and ω_+_, respectively, using eq 5
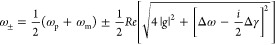
5with Δω = ω_p_ – ω_m_ and Δγ = γ_p_ – γ_m_.^46^ The resulting coupling strength (*g*) and dispersion curve for the different layer thicknesses are plotted in Figure 4a,b, respectively. Notably, as the thickness of CoPc increases, g increases from 7 to 14 cm^–1^. At 20 nm, 2 g reaches to 20 cm^–1^, which is larger than the average of full-width-at-half-maximum of CoPc (13 cm^–1^) and SPhP (22 cm^–1^) (see Supporting Information, Section S7), and satisfying the strong coupling criteria, i.e., *g* ≥ |γ_m_ + γ_p_ |/4.^44−46^ It has been shown in previous experimental and theoretical works, that the s-SNOM signal of strongly coupled systems exhibits the same Rabi splitting feature and thereby the same strong coupling criteria apply here.^46^ The coupling strength increases but plateaus near 50 nm, suggesting that molecules beyond this length do not strongly interact with the SPhP mode. The detuning dependence (Figure 4b) illustrates the expected anticrossing characteristics of polaritons (fitting and parameters shown in Supporting Information, Section S7), confirming the emergence of polaritonic states in our system.

Based on the coupling strength variation as a function of CoPc thickness, we further extracted the field distribution. First, we derived the relationship (see Supporting Information, Section S3) between the layer thickness (*d*) and the coupling strength (*g*) as
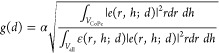
6where α is the scaling factor, *d* is the film thickness, ε = 2.7 in the volume (*V*_CoPc_) occupied by CoPc, and ε = 1 in the remaining area outside of the pillar occupied by air (see Supporting Information, Section S5), here only volume (*V*_all_) outside of the pillar is used to calculate *g*, the coupling strength, since the field strength inside of the pillar is negligible. Because all parameters are known, except the electric field profile *e*(*r*,*h*;*d*), by measuring *g*(*d*) experimentally, we can obtain *e*(*r*,*h*;*d*), if a general analytical formula of *e*(*r*,*h*;*d*) is known. Based on the field profile of the MEEP simulation, we found that a double modified Bessel function (eq S18) can accurately describe *e*(*r*,*h*;*d*) (Supporting Information, Section S3). Thus, now the field at any thickness is retrievable.

We used the general analytical expression of *e*(*r*,*h*;*d*) to calculate the coupling strength at any thickness, based on eq 6, shown as a solid line in Figure 4a. The normalized electric field at different thicknesses *d* was then obtained using *e*(*r*,*h*;*d*) (eqs S13 and S20) and plotted in Figure 4c, showing the experimental field decay with a penetration depth of ∼140 nm and discontinuity at the interfaces. This is in a good agreement with the simulated rounded edge field decay (Figure 4c). As can also be seen in this figure, we found that the pillar with a round edge (*r* = 50 nm, dashed red) exhibits a field distribution that matches much better with the experimental results when compared to the one with sharp edges (dashed black line), agreeing with the round edge detected experimentally by AFM (Figure S1). Experimentally, we did not observe an increase in Rabi splitting beyond 50 nm layer thickness due to the decrease of the averaged field with an increase of deposition thickness, compensating the  increase in Rabi splitting.

Notably, it is natural to question whether the tip of the s-SNOM strongly couples to the molecules actively and significantly changes the spectra here. We argue that based on the experimental data, the tip alone should not strongly couple to the molecules; otherwise, we should expect to see either enhanced nanophotonic signal or signature of strong coupling in places other than the edge (position 4 in Figures 2 and 3). To further elucidate this effect, a COMSOL simulation of the 3D pillar and the tip was performed (Supporting Information, Section S4). It was found that although the presence of the tip enhanced the local field by 4 times (Figure S15), it did not modify the spectral line shape (Figures S13 and S14). Thus, both the small volume under the tip with the enhanced electromagnetic field and the large volume of the entire rim of the pillar should be considered contributing to the strong coupling phenomenon.

To calculate the number of molecules involved in the strong coupling, we numerically determine the boundary of the mode volume from the simulation based on including all area with a field amplitude >1/*e* of the maximum field amplitude (field of the tip area); then, we calculate the volume inside the boundary. The volume that overlaps with the molecular layer was considered. Based on the observed strong coupling at a minimum layer thickness of 20 nm and the volume obtained by the described treatment in the paragraph above, we estimate the measured probing volume to be 4.5 × 10^4^ nm^3^ for VSC. Taking into account the lattice constant of the monoclinic β-CoPc (*a* = 1.46 nm, *b* = 0.479 nm, *c* = 1.94 nm and β = 120.78°),^58^ the volume per molecule is calculated as 1.17 nm^3^. As a result, the number of measured molecules for strong coupling is around 3.8 × 10^4^. Alternatively, by analyzing the fit in Figure 4a, we can determine the minimum coupling strength required for strong coupling to be *g*_min_ = 9.25 cm^–1^ (see Supporting Information, Section S3), based on the condition *g* ≥ | γ_m_ + γ_p_ |/4.^44,46^ Using this information, we calculate that a minimum layer thickness of 17 nm is necessary to achieve strong coupling (see Supporting Information, Section S3), leading to an estimated minimum of 3.2 × 10^4^ molecules.

We note that our estimation is larger than the number of molecules estimated previously by Wang and colleagues.^44^ A notable difference between these works is that in our study, the thickness of the molecular layer for reaching strong coupling is 17 nm, whereas in the previous work, it was concluded that only a monolayer is necessary to reach VSC, reported through a simulation of an ideal quartz pillar with a perfect edge. Such an optimized condition may be achieved when there are no defects in the pillar fabrication. However, the quality of nanofabrication can vary among different laboratories. Thus, we believe that realistically, and in our case, due to the imperfection in nanophotonic fabrications, such as a rounder edge of the pillar, it reduces the absolute enhancement from the nanophotonic structures but also extends the field penetration depths. As a result, a thicker molecular layer may be required to reach strong coupling, as reported here. However, the local hot spot under the s-SNOM tip helps to reduce the total number of molecules for strong coupling. Therefore, we see the key in being able to do experiments with a number of molecules at the order of 10^4^ in the high spatial resolution and the field enhancement through the tip.

The small effective probing volume enabled by the high-resolution technique and the local field enhancement ensures the small N limit to be reached, closer to the N that can be computationally simulated. The high-resolution techniques for accounting for the inhomogeneous field and vibrational polariton distribution in nanophotonic materials are critical, making significant progress in reaching VSC at the small *N* limit.

## Conclusions

We have demonstrated a novel approach for studying VSC at the small *N* limit by using high-resolution s-SNOM spectroscopy. Our findings reveal that field enhancement and Rabi splitting are predominantly confined to the edge of the quartz pillar, highlighting the importance of this region in achieving strong coupling. Furthermore, we have shown that high-spatial resolution spectroscopy and local field enhancement enable the selective excitation of specific modes to achieve large Rabi splitting, allowing for the targeted probing of molecules while minimizing the influence of dark modes. We find the layer thickness necessary for achieving strong coupling to be 17–20 nm rather than less than a monolayer, as reported from a previous work. We show that the discrepancy is due to the small difference in geometry consideration, i.e., a rounder photonic pillar (in our case) versus a perfect pillar with sharp edges, and the former leads to a reduction of the field enhancement and an increase of the field penetration depths, both leading to the larger molecular film thickness. Because photonic fabrications are highly dependent on the detailed parameters of instruments and other conditions, we believe that considering these small geometric changes of photonic structures could be important. Luckily, the procedure here (by combining s-SNOM scan, a series of measurements, and the numerical and analytical analysis of the results) presents a robust way to measure the field distribution experimentally, which already accounts for the imperfection in photonic fabrications.

By controlling the size and geometry of the system, we propose that the chemical properties of molecules can be fine-tuned at an ultracompact level. This is because there are fewer dark modes (e.g., 10^4^ in this case versus 10^10^ in a Fabry–Pérot microcavity) and stronger electromagnetic fields. As a result, both the LP and UP states play a more significant role in the system. The strong coupling at small *N* limit introduces new possibilities for manipulating and controlling molecular interactions on a nanoscale platform to synergize experimental and simulation works.

This research opens up opportunities for further manipulating and understanding the dynamics of strong coupling on an ultrafast time scale. Given the visible absorption of CoPc, this system holds great promise for investigating strong coupling dynamics at ultrafast time scales.

## Experimental Methods

### Quartz Pillar Fabrication

Micropillars ranging from 1 to 2.5 μm in diameter were fabricated from polished z-cut quartz crystals purchased from MTI corporation. After the crystals were cleaned, the positive photoresist AZ1505 from Microchemicals was spin-coated and soft baked at 100 °C for 60 s. The lithography exposure was done with a Heidelberg MLA150 at 375 nm. The resist was then hard baked at 100 °C for 60 s and developed in AZ400 (diluted 4:1) for 30 s. After development, the sample was plasma edged with argon at 50 mTorr and 100 W for 10 s to remove remaining contamination and improve adhesion. 200 nm of Cr were deposited by magnetron sputtering with a Denton Discovery 18. The photoresist was removed by soaking the sample in acetone for 30 min and washing residuals off with acetone and isopropanol. The sample was then dried with nitrogen. The pillars were reactive ion etched with a PlasmaPro 80 RIE from Oxford Instruments. To achieve vertical side walls with a height of 1.3 μm, this was done by etching 3 times 9 min 20 s with CF4 at 300 W, 30 mTorr, and 40 sccm flow rate as well as breaks of 5 min after each etching step to give the surface time to cool off. During the breaks, a vacuum was pumped for 30 s, followed by 4 min of purging with Ar and 30 s of pumping a vacuum. A significant improvement of the angle of the pillar was observed by this step etching process compared to etching for 28 min without breaks. After the RIE process, the Cr was removed by wet etching in chromium etchant from Microchemicals for 1 h. The sample was washed with water, acetone, and IPA to remove residuals, followed by being blown dry with nitrogen. The pillars were then inspected with a microscope to ensure that all of the chromium had been removed. The pillars with cobalt phthalocyanine (CoPc) were then placed in ultrahigh vacuum (10^–8^ Pa) to deposit the metal organic molecule (Sigma-Aldrich) with a Knudsen cell for organic molecules (CreaTec Fischer & Co. GmbH) at 330 °C. As the field enhancement is primarily on the side of the pillar, the deposition was done at an angle of 45° under constant rotation to get a homogeneous film on all sides of the pillars. Afterward, the samples with molecules were stored in dark environment to avoid degradation.

### s-SNOM Measurement

The measurements were performed at Los Alamos National Lab, with a commercial scattering-type scanning near-field optical microscope setup (Neaspec/Attocube) with PtIr coated AFM-tips (Brand: Neaspec, Model: s-SNOM), and the illumination was done with a pulsed mid-IR laser (FemtoFiber Pro, Toptica). Each spectrum was corrected by a background obtained on Si to account for the Gaussian distribution of light intensity. The spectral resolution is 6.25 cm^–1^. More details regarding the s-SNOM measurement can be found in Supporting Information, Section S5.

### OPTIR Measurement

OPTIR was performed at UCSD using a mIRage microscope (Photothermal Spectroscopy Corporation), controlled by the PTIR Studio software. The quantum cascade laser enabled measurements in the range from 920 to 1800 cm^–1^. The spectra were recorded with 50% pump power and 2.5% probe power.

### MEEP^51^ Finite-Difference Time Domain Calculation

Since the frequencies and line widths of the longitudinal and transverse optical phonon modes of the anisotropic α-quartz crystal were available in the literature,^59,60^ no additional ellipsometry experiment was performed and the available parameters were implemented in MEEP to reconstruct the anisotropic dielectric matrix, describing α-quartz during the simulation. Considering the circular symmetry of the pillar, cylindrical coordinates were used along with the absorber material boundary to avoid convergence problems associated with the absorption of transmitted/scattered light in the perfectly matched layers (PML) boundary. As in experiments, the light source is linearly polarized, we used *e*^*i*ϕ^ (*E*_r_ + *iE*_ϕ_) light source. From multiple runs of the system, we found that the space and time resolution of 10 and 10 nm/c (∼3.3 fs) gives results without signal artifact. With this, we measured reflectance and transmittance spectra of the air-quartz interface with no pillar and the quartz pillar at different geometry. Also, we collected *E*_r_, *D*_r_, *E*_ϕ_, and *D*_ϕ_ field data around the pillar. Angular fields (*E*_ϕ_ and *D*_ϕ_) are found to be much weaker compared to radial fields, which is expected, as the material is symmetric along the circle. To convert the field from a cylindrical coordinate to a Cartesian coordinate, we use the phase relation of the light source (*e*^*im*ϕ^, *m* = 1) and the fact that the material is uniaxial along the polar coordinates to obtain the corresponding field data, *E*_*x*_ = 1/2 [*e*^*i*ϕ^ (*E*_r_ + *iE*_ϕ_)+ *e*^–*i*ϕ^ (*E*_r_ – *iE*_ϕ_)] for the pillar (Figure 1a). We found that all fields are mostly confined on the edges of the quartz pillar and then decay exponentially with radius (Figure 4c,d and Supporting Information, Section S2).

### COMSOL Finite-Difference Time Domain Calculation

The pillar was simulated in COMSOL with fused SiO_2_ instead of alpha SiO_2_ as the PML layers for alpha-quartz caused the simulation not to converge. This causes a larger loss rate of the resonator, but the main physical properties remain unchanged, and therefore, this treatment was used to check the influence of the tip. The resonator was simulated with a diameter of 1.6 μm and a height of 1.3 μm; furthermore, a 20 nm rounded edge was implemented together with a 50 nm difference in radius between the top and the bottom of the pillar to match the slight cone shape of the fabricated pillar. The tip was placed 30 nm above the surface and simulated as a platinum rod with a 20 nm semispherical apex, 1 μm length, and a 16° half angle.

## Supporting Information Available

Quartz pillar AFM measurement, FDTD simulation, derivation of eqs 4 and 6, CoPc thin film spectra and permittivity determination, and coupling fitting parameters (PDF)
