# Supplementary material for: Spatially Resolved Near Field Spectroscopy of Vibrational Polaritons at the Small N Limit
Source: ACS Photonics. 2024 Jun 20;11(7):2650–8. doi: 10.1021/acsphotonics.4c00345 (PMC11258779; doi:10.1021/acsphotonics.4c00345)
Supplement: Supplementary file 1 — ph4c00345_si_001.pdf [file ph4c00345_si_001.pdf]

Supplemental Information

Spatially-resolved near field spectroscopy of vibrational polaritons at the small N limit

Oliver Hirschmann[1], Harsh H. Bhakta[1], Wilton J.M. Kort-Kamp[2], Andrew C. Jones[3], and Wei Xiong[1,3,4]

[1]Department of Chemistry and Biochemistry, University of California San Diego, La Jolla, CA 92093, USA

[2]Theoretical Divison, Los Alamos National Laboratory, Los Alamos, NM 87545, USA

[3]Center for Integrated Nanotechnologies, Materials Physics and Applications Division, Los Alamos National Laboratory, Los Alamos, NM, USA

[4]Materials Science and Engineering Program, University of California San Diego, La Jolla, CA 92093, USA

[5]Department of Electrical and Computer Engineering, University of California San Diego, La Jolla, CA 92093, USA

Content

1. Quartz pillar fabrication and thin film deposition 3
Quartz pillar height measurement 3
Cobalt phthalocyanine thin film deposition 3
2. FDTD simulation 4
Quartz pillar resonator simulation 4
Quartz pillar resonator spectrum 7
Quartz pillar simulated field extension (in air) 8
Quartz pillar simulated field extension (with molecular layer) 9
3. Fitting derivation 11
Harmonic oscillator fitting model 11
Coupling strength derivation 12
Analytical Expression of the electric fields 14
Field decay and penetration depth simulation vs. experiment 16
Minimum strong coupling thickness 16
4. COMSOL simulation to determine tip influence 16
Estimating the number of coupled molecules based on COMSOL calculation 18
5. CoPc thin film 19
Extraction of CoPc permittivity 19
Cobalt phthalocyanine thin film measured with OPTIR 21
Cobalt phthalocyanine thin film measured with s-SNOM 21
6. s-SNOM 22
7. Pillar resonator and CoPc coupling fitting parameters 23
8. References 29

1. Quartz pillar fabrication and thin film deposition

Quartz pillar height measurement

After etching the pillar and taking off the hard mask, the fabricated structures were measured with atomic force microscope (AFM). A representative line scan showing the height (1.3 μm) of the pillar is presented in Figure S1. The right side shows an artifact of the tip, as during scanning, the tip cannot follow the nearly vertical profile of the pillar due to its shape.

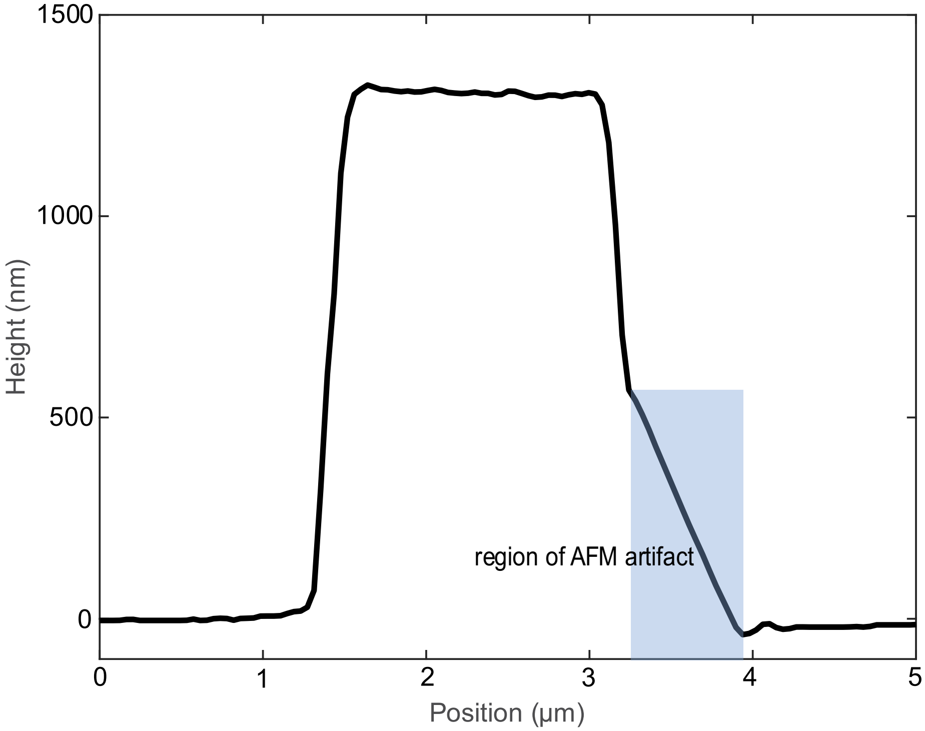

Figure S1. Quartz pillar resonator height measurement with AFM. The marked region on the right side is an artefact due to the shape of the AFM tip. It cannot follow the almost vertical profile of the pillar due to its shape.

Cobalt phthalocyanine thin film deposition

To fabricate a thin film that not only covers the top of the pillar but also the side of it, we deposited the Cobalt phthalocyanine (CoPc) vapor at an angle of 45°. To further ensure a homogeneous coverage on all sides of the pillar, the quartz crystal was continuously rotated during deposition.

A reliable thin film thickness measurement on the side of the pillar is very challenging if doable at all. So, we measured the CoPc thickness deposited on a flat quartz crystal (figure S2b) by keeping the same deposition angle (45°) and rate as the deposition to the rotating pillar, which reflect the deposition cross section projected onto a tilted

piece (figure S2c). Because the pillar is rotating, it effective makes the deposition area to be π times of the deposition cross-section. Based on that the materials deposited at a given time is the same, the thickness of the rotating pillar needs to be $\frac{1}{\pi}$ of the one of flat piece to compensate the area difference.

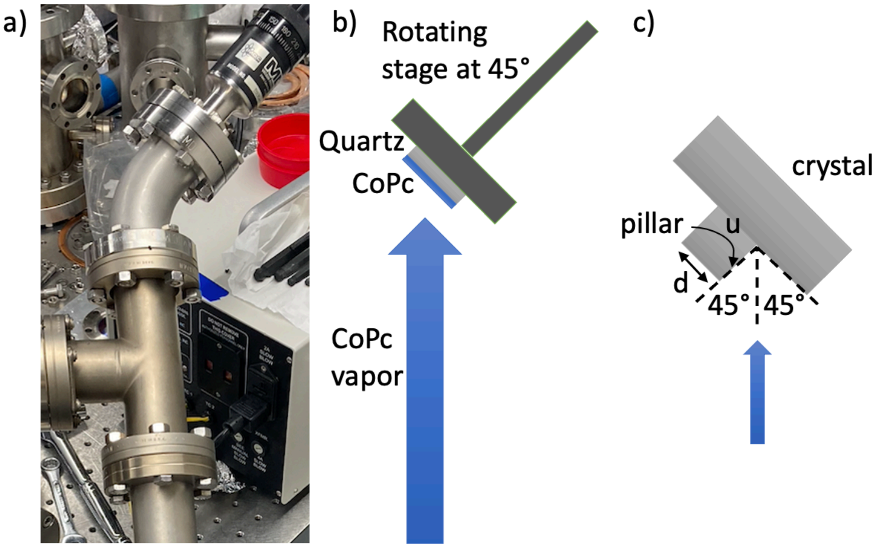

Figure S2. Setup for the thin layer deposition of Cobalt phthalocyanine including a rotating stage at 45°. (a) showing the actual setup with the Knudsen cell inside the tube and the 45° angle on top. (b) a schematic drawing of the CoPc vapor deposition on the quartz crystal at the angle. (c) showing the deposition at 45°, leading to a similar deposition rate on the surface of the crystal as well as the side of the pillar.

2. FDTD simulation

Quartz pillar resonator simulation

The quartz resonator confines the electromagnetic field in a small mode volume limited to the top corner edge of the pillar. This strong confinement in a small volume is a key to achieving strong coupling with only a few thousand molecules. Figure S3 shows the E_x field profile from the side view of the pillar with perfect edges, while Figure S4 shows the amplitude of the E_x field on log scale, showing that the area around the edge of the perfect pillar has most of the confined field. The field profiles were simulated using MEEP program (details in Method section).

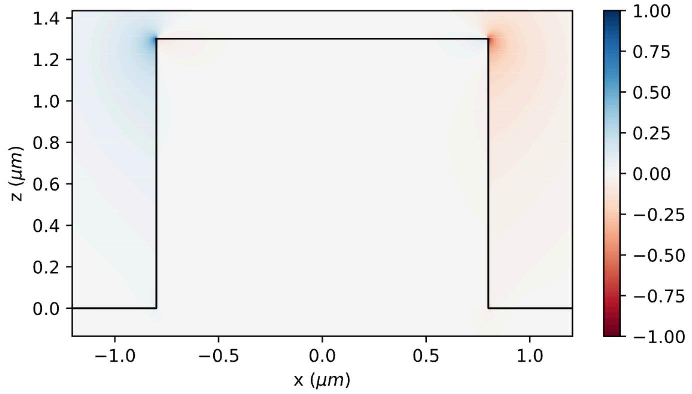

Figure S3. Side view of the simulated E_x field distribution for a 1.6 μm in diameter and 1.3 μm in height quartz pillar, showing confinement on the edge.

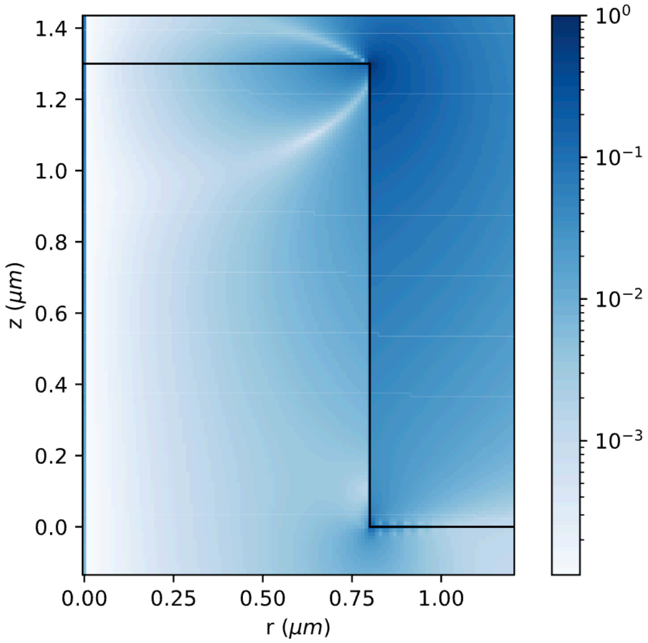

Figure S4. Zoomed-in side view of the 1.6 μm in diameter and 1.3 μm in height quartz pillar, showing amplitude of E_x field with the high confinement on the edge.

While the pillar with perfect edges (figure S3 and S4) shows large confinement, due to imperfect fabrication in experiments, the edge of the fabricated pillar is slightly rounded (figure S1), leading to less confinement on the edge. The enhancement was therefore calculated with a rounded edge of 50 nm radius, as shown in figure S5 and S6. The displaced field was normalized with respect to the field on top of a flat quartz substrate for S5 and S6 and with respect to vacuum for figure 2a. A comparison of the confinement between the pillar with a perfect edge and a rounded edge is provided in

figure S7, concluding that the rounded edge pillar simulation results agree with the experimental data better.

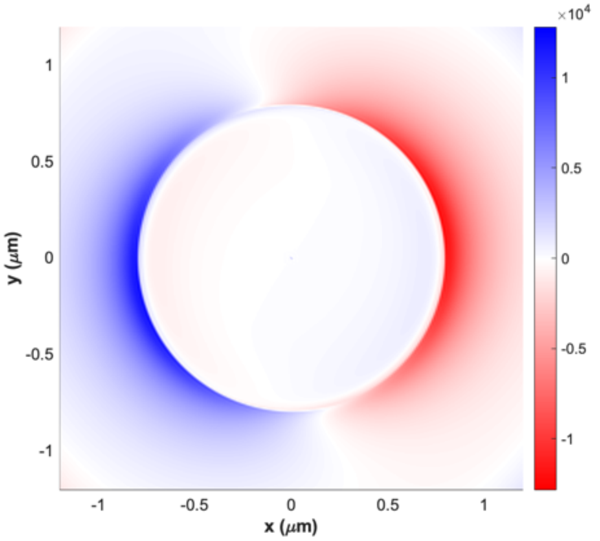

Figure S5. Quartz pillar resonator with 50 nm rounded edge radius top view, enhancement normalized to a flat quartz surface.

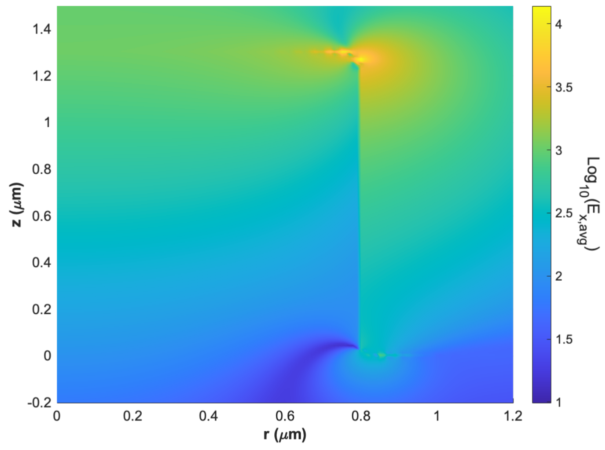

Figure S6. Quartz pillar resonator with 50 nm rounded edge side view, enhancement normalized to a flat quartz surface, logarithmic scaled.

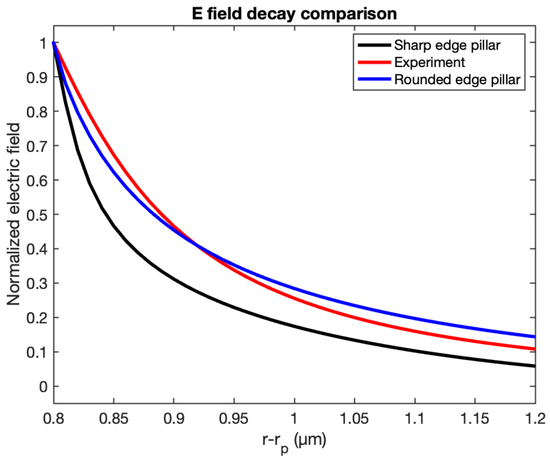

Figure S7. Comparison between the simulated radial extension of E_x-field amplitude for a 1.6 μm in diameter and 1.3 μm in height quartz pillar with a sharp edge (black) and a 50 nm rounded edge (blue), plotted in comparison with the experiment extracted decay (red).

Quartz pillar resonator spectrum

In addition to the spatial simulation, the reflectance spectrum of the resonator was also simulated, shown here (Figure S8). The frequency positions of the simulated peaks are in a good match and especially the main peak matches well with the experimental peak observed at 1129cm^{-1} (Figure 2).

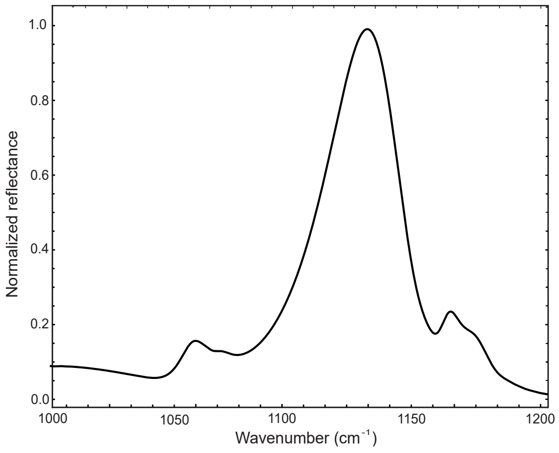

Figure S8. Simulated reflectance spectrum of a 1.6 μm in diameter and 1.3 μm in height quartz pillar, normalized with respect to the reflectance spectrum of a quartz surface with no pillar.

Further, the spectra at different spatial position around the pillar were simulated for a pillar of 1.6 μm diameter and 1.3 μm height with a 50 nm rounded edge radius and

shown in figure S9a, with comparison to the experimental spectra of figure 2d included. A similar comparison was shown in figure S9b with the pillar covered by a 20 nm CoPc layer. In both figures of figure S9, the central peaks around 1130 cm^{-1} in blue region fits the spectra well, validating the simulation approach to describe resonance in this region. However, it is not the case for lower frequency peaks around 1070 cm^{-1}. This might be due to the following reasons: the MEEP simulation was discrete with a resolution of 10 nm, which resulted into poor rounded structure and thereby giving extra artifacts; and only the in-plane modes can be observed in the MEEP simulation, as restricted by the cylindrical coordinates and parallel wave method used in the simulations. Regardless, because the 1070 cm^{-1} peak does not involve in strong coupling, it is out of the scope of the present work.

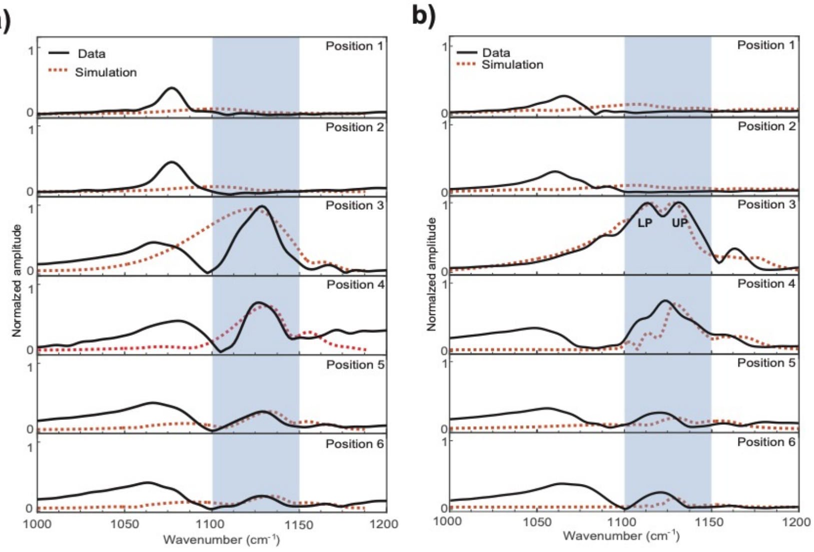

Figure S9. Simulated position dependent amplitude spectrum of a 1.6 μm in diameter and 1.3 μm in height quartz pillar (a), covered with a 20 nm CoPc layer (b).

Quartz pillar simulated field extension (in air)

To visualize and quantify the confinement of the resonator, the field intensity was extracted from the simulation (10 nm resolution) with the rounded edge, normalized and plotted versus the radial distance from the surface of the pillar (shown in blue in Figure S10). The values were then fitted with a modified Bessel function K_1 ($\boldsymbol{y = a_r * \frac{K_1(\,b_r*(x+0.8))}{\sqrt{(x+r_r)^2+h_r^2}}}$) to quantify the decay.[1] The fitted values are $\boldsymbol{a_r = 0.13\ \mu m}$, $\boldsymbol{b_r = 0.85\ \mu m^{-1}}$, $\boldsymbol{h_r = 0.001\ \mu m}$, $\boldsymbol{r_r = 0.08\ \mu m}$. With the field penetration depth $\boldsymbol{\tau_r}$ being $\boldsymbol{y(\tau_r) = \left(\frac{y(0)}{e}\right)}$,[2] the value for the radial-penetration depth of the pillar in air is 150 nm. We also used a Bessel function for the experimental fit (Figure 4a, c) for the radial decay.

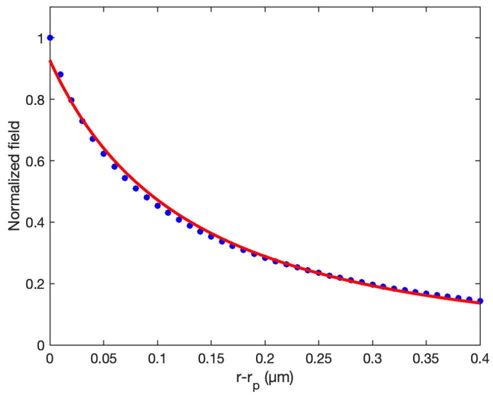

Figure S10. Simulated radial extension of E_x-field amplitude for a 1.6 μm in diameter and 1.3 μm in height quartz pillar with a 50 nm rounded edge, plotted beyond the surface (blue) and fitted with a Bessel (red).

A comparable approach was taken for the height-field decay (S11), down along the height from the top edge of the pillar, the normalized simulated field intensity was fitted empirically with ($\boldsymbol{y = a_h(\frac{1}{x+b_h})}$) to quantify the decay. The fitted values are $\boldsymbol{a_h = 0.13\ \mu m,\ b_h = 0.14\ \mu m}$. With the field penetration depth $\boldsymbol{\tau_h}$ being $\boldsymbol{y(\tau_h) = \left(\frac{y(0)}{e}\right)}$, the value for the height-penetration depth from the edge of the pillar is 210 nm.

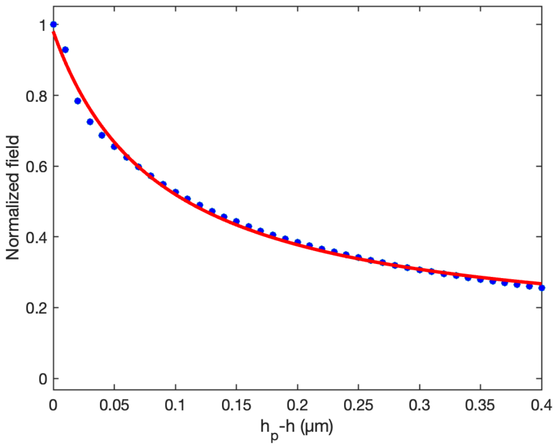

Figure S11. Simulated height extension of E_x-field amplitude for a 1.6 μm in diameter and 1.3 μm in height quartz pillar, plotted as field extension on the surface, with 0 μm being the top of the pillar. The simulated data (blue) was fitted with a biexponential (red).

Quartz pillar simulated field extension (with molecular layer)

The quartz pillar, covered with a 300 nm molecular layer was simulated with MEEP to investigate the decay and continuity of the electric field. The electric field was found

to be non-continuous between the molecular layer and air for E_x, as shown in figure S12. Figure S12 further shows the decay and continuity for the different E and D fields (E_x, D_x, E_r, D_r, and E_ϕ, D_ϕ). An analytical form to describe the field distribution of the quartz pillar covered by molecular layers is derived in the next section.

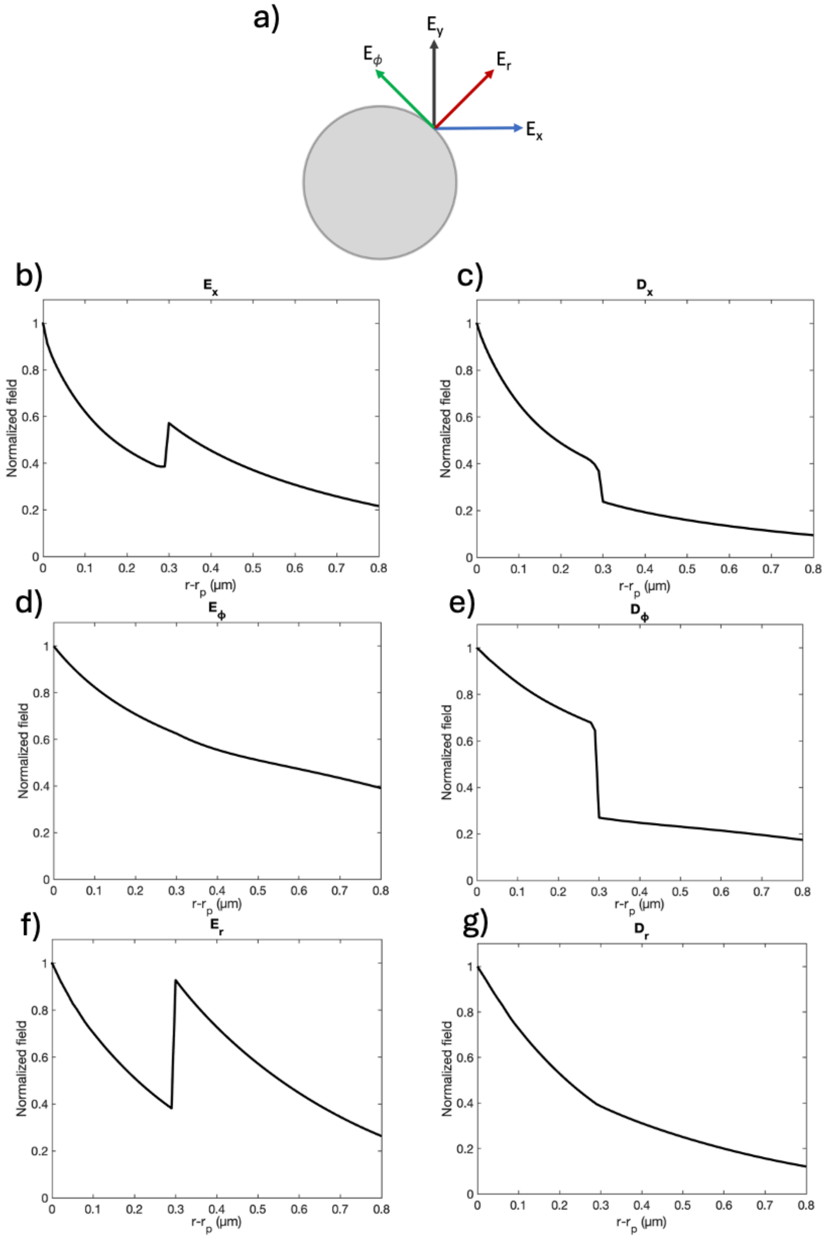

Figure S12. Simulated radial extension of the field amplitude (E_x, D_x, E_r, D_r, and E_ϕ , D_ϕ), for a 1.6 μm in diameter and 1.3 μm in height quartz pillar with a 50 nm rounded edge, covered with 300 nm of CoPc, plotted beyond the surface. The trace shows the non-continuity between the CoPc layer (0-0.3 μm) and air (>0.3 μm) for E_x.

3. Fitting derivation

Harmonic oscillator fitting model

For modeling the coupling of the CoPc and quartz pillar, we used two coupled classical harmonic oscillators (x_m and x_p), driven by the external force (F_m and F_p, respectively).[3]

$$\ddot{x}_p(t) + \gamma_p \dot{x}_p(t) + \omega_p^2 x_p(t) - 2g\dot{x}_m(t) = F_p(t) \tag{S1}$$

$$\ddot{x}_m(t) + \gamma_m \dot{x}_m(t) + \omega_m^2 x_m(t) + 2g\dot{x}_p(t) = F_m(t) \tag{S2}$$

Using the Fourier-transform of the above equations, we solved for the steady state solution of the coupled oscillator.

$$-x_p(\omega)\,\omega^2 - i\omega\gamma_p x_p(\omega) + \omega_p^2 x_p(\omega) + 2i\omega g x_m(\omega) = F_p(\omega) \tag{S3}$$

$$-x_m(\omega)\,\omega^2 - i\omega\gamma_m x_m(\omega) + \omega_m^2 x_m(\omega) - 2i\omega g x_p(\omega) = F_m(\omega) \tag{S4}$$

Resulting into the solution for x_p and x_m,

$$x_p(\omega) = \frac{(\omega_m^2-\omega^2-i\gamma_m\omega)F_p(\omega) - 2i\omega g\, F_m(\omega)}{(\omega_m^2-\omega^2-i\gamma_m\omega)(\omega_p^2-\omega^2-i\gamma_p\omega)-(2g\omega)^2} \tag{S5}$$

$$x_m(\omega) = \frac{(\omega_p^2-\omega^2-i\gamma_p\omega)F_m(\omega) + 2i\omega g\, F_p(\omega)}{(\omega_m^2-\omega^2-i\gamma_m\omega)(\omega_p^2-\omega^2-i\gamma_p\omega)-(2g\omega)^2} \tag{S6}$$

Since the external illumination is weak compared to the resonator, we keep $\mathbf{F_m = 0}$. Further, it is worth mentioning that spectral fitting with $\mathbf{F_m \neq 0}$ showed no improvement in fitting (comparison shown in section 7, figure S20). The measurement in s-SNOM involves detection of both the amplitude and phase of the field.[3] Since the amplitude is comparable to the absorbance spectra, the absorbance of light was calculated as product of $\dot{x}_p$ and F_p averaged over the period.[4]

$$C \propto \langle \dot{x}_p(t) F_p(t) \rangle \Rightarrow C(\omega) \propto \omega\, Im\left(x_p(\omega) F_p^*(\omega)\right) \tag{S7}$$

$$C(\omega) \propto \omega\, Im\left(\frac{\left((\omega_m^2-\omega^2-i\gamma_m\omega)|F_p(\omega)|^2\right)}{(\omega_m^2-\omega^2-i\gamma_m\omega)(\omega_p^2-\omega^2-i\gamma_p\omega)-(2g\omega)^2}\right) \tag{S8}$$

Following Dolado et al., to account for the drift in the interferometric measurement between the reference silicon and the CoPc covered quartz resonator, we included an extra phase shift ϕ into the surface-phonon mode (x_p).[3]

$$x_{exp}(\omega) = x_p(\omega) e^{i\phi} \tag{S9}$$

$$C_{exp}(\omega) \propto \omega\, Im\left(\frac{\left((\omega_m^2-\omega^2-i\gamma_m\omega)\left|F_p(\omega)\right|^2\right)e^{i\phi}}{(\omega_m^2-\omega^2-i\gamma_m\omega)(\omega_p^2-\omega^2-i\gamma_p\omega)-(2g\omega)^2}\right) \quad \text{(S10)}$$

Thus, using eq. S10, we can fit the measured spectra for different pillar diameters and different thickness of deposition (see all fitting results in figure S22-25 and table S5-8) and extract the individual coupling strength g, cavity (molecular) mode $\omega_p(\omega_m)$ and dissipation $\gamma_p(\gamma_m)$, and force F_p parameters. These parameters were then used in eq. 5 to calculate the eigenfrequencies $\omega_\pm$ as shown in figure 4b, and the points were the fitted with the eq. 5 to obtain the fitted coupling strength *g* corresponding to each deposition thickness (figure 4a-b).

Coupling strength derivation

In the quartz pillar resonator system, the CoPc molecules deposited on the pillar are not coupled to the polariton mode of the pillar in a uniform manner, i.e., the coupling of different molecules is different based on the electric field of the confined mode at their position. As the regular Tavis-Cummings model assumes the electric field being constant for all the oscillators, which is not the case here, we instead used the following modified Tavis-Cummings model, which allows for the different coupling constant for different molecules.[5,6]

$$\hat{H} = \omega_c a^\dagger a + \sum_i^N \left(\omega_o \hat{\sigma}_i^\dagger \hat{\sigma}_i + g_i\left(a^\dagger \hat{\sigma}_i + h.c.\right)\right) \quad \text{(S11)}$$

Here, ω_c (ω_o) is the frequency of the cavity (vibrational) mode, $a^\dagger(a)$ and $\hat{\sigma}_i^\dagger(\hat{\sigma}_i)$ are the creation (annihilation) operators for the cavity and vibrational modes (of the ith molecule), respectively. The operators for vibrational modes can be represented in the following orthonormal representation.[5]

$$\hat{H} = \omega_c a^\dagger a + \omega_o \hat{S}_o^\dagger \hat{S}_o + G_N\left[\hat{a}\hat{S}_o^\dagger + \hat{a}^\dagger \hat{S}_o\right] + \omega_o \sum_j^{N-1} \hat{S}_j^\dagger \hat{S}_j \quad \text{(S12)}$$

Where, $G_N = \sqrt{\sum_i^N g_i^2}$ is the net coupling strength for **N** number of oscillators, $\hat{S}_o^\dagger = \frac{1}{G_N}\sum_{i=1}^N g_i \hat{\sigma}_i^\dagger$ is the creation operator for bright mode, and $\hat{S}_j^\dagger = \frac{g_{j+1}}{G_j G_{j+1}}\sum_{i=1}^j g_i \hat{\sigma}_i^\dagger - \frac{G_j}{G_{j+1}}\hat{\sigma}_{j+1}^\dagger$ is the creation operator for the dark modes.[5] The bright mode part of the Hamiltonian (the first three terms of S12, without dark mode terms $\hat{S}_j$) can be recognized as similar to Jaynes-Cumming model with $\mathbf{g} \rightarrow G_N$, and $\sigma_o \rightarrow \hat{S}_o$ replacing, thereby has the same solutions as Jaynes-Cumming model but with replaced terms. Thus, the **g**

value obtained from the fitting in the previous section, Harmonic oscillator fitting model, (with results in section 7) is equivalent to **G_N** for the quartz-pillar resonator.

Now, **g_i** for molecules at different position of the pillar is different, i.e., $\boldsymbol{g_i} = |\boldsymbol{\mu}(\boldsymbol{r_i}, \boldsymbol{h_i}, \boldsymbol{\phi_i})\ .\boldsymbol{E}(\boldsymbol{r_i}, \boldsymbol{h_i}, \boldsymbol{\phi_i})|$, where **$r_i$**, ϕ_i, and **h_i** are the radius, angle, and height from the central axis of the pillar of the corresponding molecule **i**. Using the second-quantized electric field for the cavity modes in 3D[7,8], the coupling strength can be written as[9]

$$\vec{E}(\vec{r}) = \vec{e}(\vec{r})\sqrt{\frac{4\hbar\omega_c}{\varepsilon_0 \int\left(\varepsilon_g(\vec{r})+\varepsilon(\vec{r})\right)|e(\vec{r})|^2 d^3r}} \quad \text{(S13)}$$

$$g_i = |\mu(r_i, h_i, \phi_i).\vec{e}\,(r_i, h_i, \phi_i)|\sqrt{\frac{4\hbar\omega_c}{\varepsilon_0 \int\left(\varepsilon_g(\vec{r})+\varepsilon(\vec{r})\right)|e(\vec{r})|^2 d^3r}} \quad \text{(S14)}$$

Where, ω_c is the cavity mode frequency, $\vec{e}(r)$ is the mode's field distribution of the cavity mode, $\hbar$ is reduced Planck's constant, ε_0 is the vacuum permittivity, ε_g is the real part of the group permittivity $\varepsilon_g(\vec{r}) = \frac{\partial(\varepsilon(\omega,\vec{r})\omega)}{\partial\omega}$, and $\varepsilon(\vec{r})$ is the real part of the relative permittivity at position $\vec{r}$. Considering that the CoPc is treated as an oscillator coupling to the cavity, we do not treat the CoPc layer dispersively when calculating the field, so as not to double account for the oscillator field interaction. As the numerical simulation (Fig. S5-6) indicates, the field E_x is much less inside the quartz pillar compared to the outside. Thus, treating only the field that is outside of the quartz pillar, where all the CoPc and air are non-dispersive, we replace ε_g with ε for group permittivity.

Summing over the individual molecules coupling in eq. S16 and assuming homogeneous distribution of molecules, results in the net coupling strength, **G_N**, referred now as g, to simplify the notation.[10,11]

$$G_N = \sqrt{\sum_i^N g_i^2} \Rightarrow g = \sqrt{\int \rho(\vec{r}) g_i^2(\vec{r}) d^3r}$$

$$g(d) = \sqrt{\frac{2\hbar\omega_c\rho \int_{V_{CoPc}} |\vec{\mu}\cdot\vec{e}(r,h,\phi;d)|^2 r dr\, dh\, d\phi}{\varepsilon_0 \int_{V_{all}} \varepsilon(r,h;d)|\vec{e}(r,h,\phi;d)|^2 r dr\, dh\, d\phi}} \quad \text{(S15)}$$

Where, ρ is the number density of CoPc molecules, $\varepsilon(r,h;d) = \begin{cases} \varepsilon_{CoPc}, & r_p \le r \le r_p + d \text{ and } 0 \le h \le h_p + d \\ \varepsilon_{air}, & otherwise \end{cases}$,
with $\varepsilon_{CoPc} = \varepsilon_\infty$ of the CoPc given in Table S2, r_p (h_p) is radius (height) of the pillar, d is the deposition thickness of the material, and V_{CoPC}(V_{all}) is volume of the CoPc layer (all

space except inside of the quartz, respectively). Considering that the mode coupled in the simulations has mode field in in-plane direction and the light is linearly polarized, here, we use $\hat{e}_x$ as the direction of the field profile observed.

As the pillar has cylindrical symmetry, the fields in cylindrical coordinate system comprising the e_x would also be symmetric, i.e., $\boldsymbol{e_x(r,h,\phi) = e_r(r,h)\cos(\phi) - e_\phi(r,h)\sin(\phi)}$, where e_r and e_ϕ are angle independent field profile. Also, for simplification, we assume that the aggregate direction of the transition dipole moment of the homogeneous medium is independent of position, i.e., $|\vec{\boldsymbol{\mu}}\cdot\vec{\boldsymbol{e}}(\boldsymbol{r,h,\phi})| = \boldsymbol{a}|\boldsymbol{\mu}||\boldsymbol{e_x(r,h,\phi)}|$, with *a* being the constant determined by the angle between the dipole moment and field. Now, evaluating the integral in eq. S17 with respect to ϕ, we get the following.

$$\int_0^{2\pi} |e_x(r,h,\phi)|^2 d\phi = \int_0^{2\pi} \left|e_r(r,h)\cos\phi - e_\phi(r,h)\sin\phi\right|^2 d\phi$$

$$= \left(|e_r(r,h)|^2 + \left|e_\phi(r,h)\right|^2\right)\pi = 2\pi\, e_{x,rms}^2(r,h) \qquad \textbf{(S16)}$$

Where, $\boldsymbol{e_{x,rms}(r,h)} = \sqrt{\frac{|e_r(r,h)|^2+\left|e_\phi(r,h)\right|^2}{2}}$ is the root mean square averaged e_x field along the angular ϕ axis. This treatment greatly simplifies the analytical treatment for the calculation of the coupling strength, as now, we do not have to consider the angular field profile, and, instead of the separate treatment of the two profiles, e_r and e_ϕ, we combined them and give an analytical equation of one field profile, $\boldsymbol{e_{x,rms}(r,h)}$, which as we will see later, would reduce the number of unknown parameters needed to fit the experimental data.

$$g(d) = \alpha\sqrt{\frac{\int_{V_{CoPc}} e_{x,rms}^2(r,h;d)\,r dr\, dh}{\int_{V_{all}} \varepsilon(r,h;d)\,e_{x,rms}^2(r,h;d)\,r dr\, dh}} \qquad \textbf{(S17)}$$

Here, $\boldsymbol{\alpha} = \sqrt{\frac{2\hbar\omega_c\,\rho\, a^2\,|\mu|^2}{\varepsilon_0}}$ is the proportionality constant. Thus, based on experimentally measured coupling strength at different deposition thickness d (figure 4a), if we know the general analytical form of the electric field profile, $\boldsymbol{e_{x,rms}(r,h)}$, we can use (S17) to extract $\boldsymbol{e_{x,rms}(r,h)}$ and eventually the electric field strength E(r) in eq. S20.

Analytical Expression of the electric fields

The analytical form of the electric field can be obtained by generalization of the numerical simulation in Fig. S6. The field profile in the simulation is highly complex, as

such, we make certain assumptions to describe the profile in a simple manner. 1) Considering that the field inside of the quartz is much smaller (less than 10 times) compared to the outside, the analytical form assumes the field inside is zero. 2) For a cylindrical waveguide, the evanescent waves are described as a modified Bessel function (also known as modified Hankel function) of the first kind (K_1), as such we describe the decay on the side of the pillar as having the form of Bessel function.[1] 3) In figure S6, on the rounded edge of the pillar, we observe a radial-like scatter around the top edge of the pillar, which in the far-field does behave radially. Considering these assumptions and with the purpose of keeping the model accurate but simple, we analytically describe the electric field distribution as following:

$$\boldsymbol{e_{x,rms}(r,h;d)} = \begin{cases} \mathbf{0}, & r<r_p \text{ or } h<0 \\ \frac{K_1(b_{CoPc}\, r)}{\sqrt{(r-r_c)^2+(h-h_c)^2}}, & r_p \le r \le r_p+d \text{ and } 0 \le h \le h_p+d \\ c(d)\, c_r \frac{K_1(b_{air}\, r)}{\sqrt{(r-r_c)^2+(h-h_c)^2}}, & Otherwise \end{cases} \quad \text{(S18)}$$

Where, $\mathbf{b}_{air}$ ($\mathbf{b}_{CoPc}$) are the average decay constant in air (CoPc) medium, $\mathbf{r}_p$ ($\mathbf{h}_p$) are the radius (height) of the pillar, $\mathbf{d}$ is the deposition thickness, ($\mathbf{r}_c$,$\mathbf{h}_c$) are the origin of the radial decay, $c(d) = \frac{K_1\left(b_{CoPc}\,(r_p+d)\right)}{K_1\left(b_{air}\,(r_p+d)\right)}$ is the constant to ensure the continuity across the interface (CoPc to air) when $c_r=1$, and $\mathbf{c}_r$ is the average discontinuity ratio across the interface of the two media, which depends on the contribution of the radial e_r and angular e_ϕ field in the $e_{x,rms}$(eq S15). For example, if only radial field contributes, then because displacement field is continuous (figure S12), the ratio c_r would be $\varepsilon_{CoPc}/\varepsilon_{air}$ =2.7, corresponding to the $\varepsilon_{CoPc}\, e(r_p+d)^- = \varepsilon_{air}\, e(r_p+d)^+$ continuity condition.

The root-mean-square angle-averaged electric field $E_{rms}(r,h;d)$ for the pillar with material deposited on it can be obtained by substituting eq S18 into eq S13.

$$\boldsymbol{E_{rms}\,(r,h;d) = e_{x,rms}(r,h;d)\sqrt{\frac{\hbar\omega_c}{\pi\varepsilon_0 \int_{V_{all}} \varepsilon(r,h;d) e^2_{x,rms}(r,h;d) r dr\, dh}}} \quad \text{(S19)}$$

And its normalized form versus $\boldsymbol{E_{rms}(r_p,h_p;0)}$, the field strength at the edge of the pillar is

$$\frac{E_{rms}(r,h;d)}{E_{rms}(r_p,h_p;0)} = \frac{c(0)e_{x,rms}(r,h;d)}{c(d)e_{x,rms}(r_p,h_p;0)}\left(\frac{\int_{V_{CoPc}} \frac{\frac{\varepsilon_{CoPc}}{c(d)c_r^2\varepsilon_{air}} K_1(b_{CoPc} r)^2 - K_1(b_{air} r)^2}{(r-r_c)^2+(h-h_c)^2} r dr dh}{\int_{V_{all}} \frac{K_1(b_{air} r)^2}{(r-r_c)^2+(h-h_c)^2} r dr dh} + 1\right)^{-\frac{1}{2}} \quad \text{(S20)}$$

Using the eq S17, coupling strength data at different deposition thickness d was fitted (figure 4b) and parameters in eq 18 were extracted from the fit. The parameters were then used in eq S20 to obtain the normalized root mean square angle-averaged electric field as can be seen in Fig 4c.

Field decay and penetration depth simulation vs. experiment

The radial decay of the simulated electric field $e_{x,rms}$, obtained by doing FDTD simulation in MEEP of the pillar (with geometry: 1.6 μm diameter, 1.3μm height, and 50 nm rounded edge radius) for various deposition thickness d, was fitted with eq S18 to obtain the decay parameters (Table S1, simulation row). No change in the decay parameters (b_{CoPc} and b_{air}) were found in the simulation when changing the layer thickness. Similar parameters were obtained through the fitting of the experimentally obtained coupling strength vs deposition thickness (figure 4a) using eq S17, where eq S18 was implicitly applied in the fitting. The agreement between the corresponding decay values for inside and outside the molecular layer (b_{CoPc} and b_{air}) as well as the discontinuity ratio (c_r) can be seen in table S1, indicating the agreement of the experimental field and the simulated field at the cavity mode frequency. Consequently, the penetration depth found by simulation (150 nm) agrees well with the one found by experiment (140 nm) (figure 4c and S7).

	b_{CoPc} (μm^{-1})	b_{air} (μm^{-1})	c_r
Simulation	1.2	0.9	1.4
Experiment	1.1	0.9	1.5

Table S1. Figure 4c simulation and experiment parameters, showing the agreement between them

Minimum strong coupling thickness

The criterium for strong coupling is defined as $\boldsymbol{g} \geq |\boldsymbol{\gamma_m} + \boldsymbol{\gamma_p}|/\boldsymbol{4}$.[3,12] The average value for $|\boldsymbol{\gamma_m} + \boldsymbol{\gamma_p}|/\boldsymbol{4}$, hence the minimum $\boldsymbol{g}$ required for strong coupling, is calculated to be $\boldsymbol{g_{min}} = \boldsymbol{9.25\ cm^{-1}}$ (Table S6 – 20nm CoPc deposition). With the fitting obtained for $\boldsymbol{g}$, the minimum layer thickness for strong coupling is calculated to be $\boldsymbol{17\ nm}$. We therefore conclude that for the system studied in this work, a minimum CoPc thickness of 17 nm is required to achieve strong coupling.

4. COMSOL simulation to determine tip influence

The pillar (1.6 μm top diameter, 1.3 μm height, 20 nm rounded edge radius, 1.65 μm bottom diameter of the cone) was calculated in 3D with COMSOL to investigate the influence of the tip on the spectra and the coupling. First, the SiO_2 pillar was simulated

in 3D. Then, the simulated metal tip (Pt, 20 nm semispherical apex, 1 µm length, 16° half angle) was placed 30 nanometers above the surface at the edge of the pillar and the simulated field enhancement was compared between with and without the tip. It can clearly be seen that the field has been enhanced locally at the position of the tip (figure S13 spectrum plotted at the surface of the pillar – amplitude: $9*10^{-4}$ without the tip (a), $2*10^{-3}$ with the tip (b)). However, the spectral distribution has not been influenced significantly. This shows that the tip causes a local enhancement in the electric field but does not cause a major change of the spectral line shape for the pillar measurement. The measurement is therefore passive in nature.

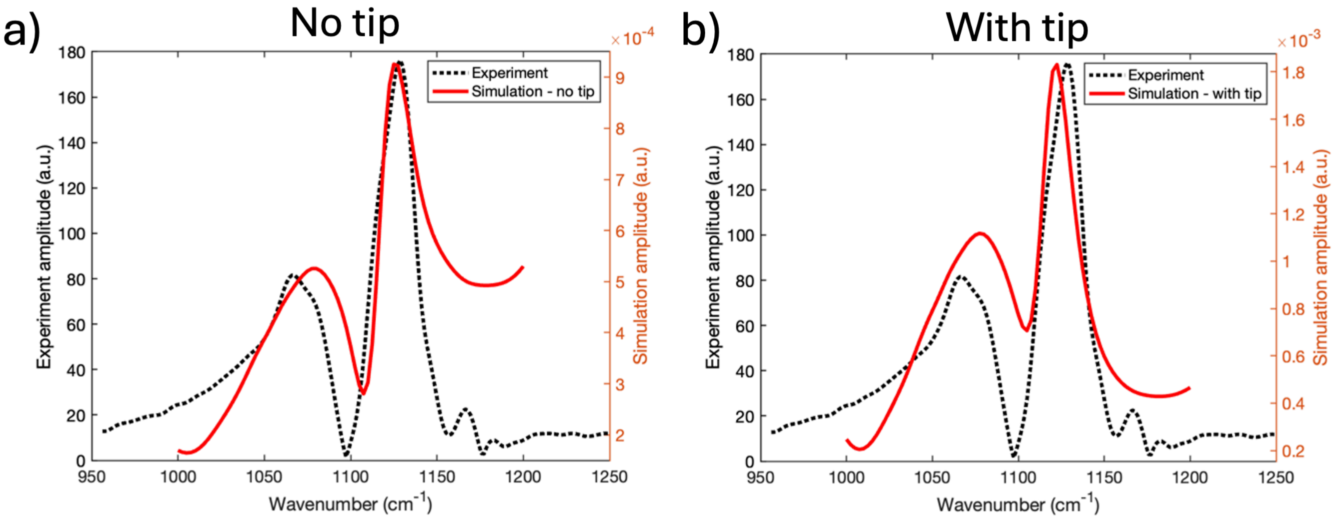

Figure S13. Resonator pillar spectrum on the edge of the pillar (figure 2c and d, position 3), without the tip (a) and with the tip (b) taken on the surface of the pillar. Except for a field enhancement, no major change is observed in the spectrum, indicating the passive role of the tip.

In addition to the comparison between the pillar and the pillar with the tip, another pair of simulations was performed with a 20 nm CoPc layer covering the pillar (Figure S14). The comparison for that case shows that through the presence of the tip, a slight change in frequency happens but again no major change in spectrum is associated to the presence of the tip. We observe a strong background signal in the simulated spectrum due to near-field scattering.

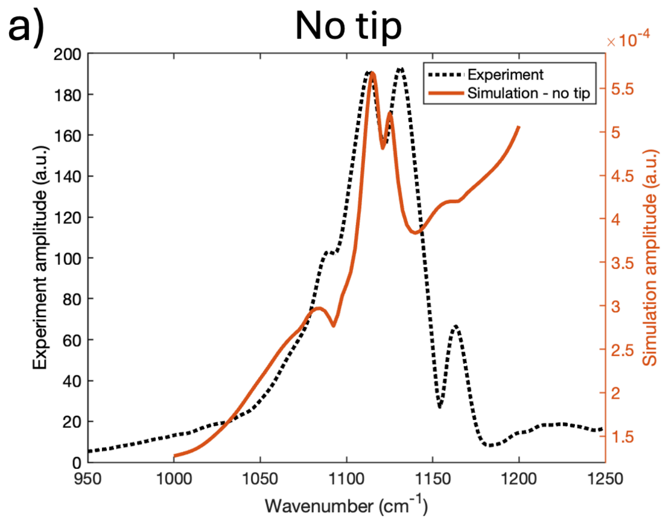

Figure S14. Resonator pillar with a 20 nm CoPc layer covering it, spectrum on the edge of the pillar (figure 3, position 3), without the tip (a) and with the tip (b) taken on the surface of the pillar. No significant change is observed in the spectrum, indicating the passive role of the tip.

The simulation in COMSOL was done with fused SiO_2 instead of alpha SiO_2 due to convergence issues. The fused silica has a larger loss rate, leading to the difference in Rabi splitting compared to the MEEP simulation and the experiment (figure S6). However, the main physical properties of the resonator remain unchanged and therefore, this treatment has been used to check the influence of the tip.

In COMSOL, the pillar was simulated with both a rounded edge and a cone shape. This was also done in MEEP, but due to the homogeneously-distributed rectangular grid in MEEP with the computationally limited resolution of 10 nm, this caused artefacts and was not further used. Instead, the MEEP results used in here had pillar with a rounded edge but no cone shape.

Estimating the number of coupled molecules based on COMSOL calculation

The number of molecules considered to be involved in the strong coupling is calculated based on the scattered electric field distribution obtained by COMSOL, including the tip. While there is no significant spectral difference with and without the tip, as explained above, 4 times enhancement of the electromagnetic field happens around the tip (figure S15).

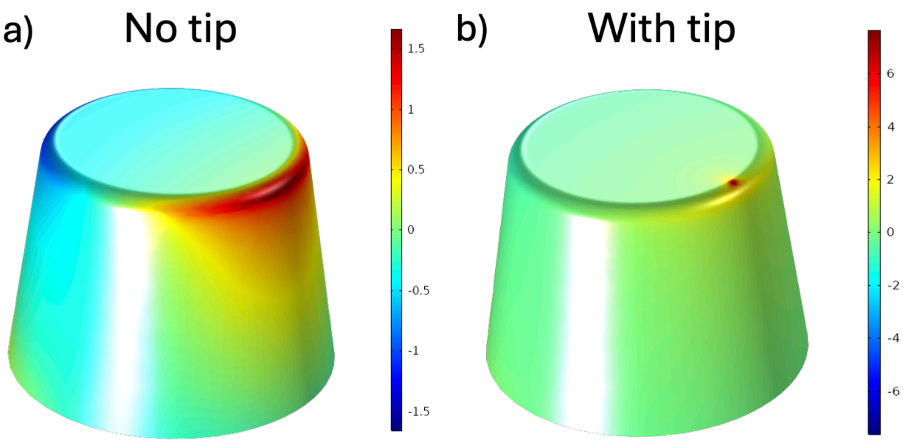

Figure S15. Resonator pillar simulated in COMSOL with no tip (a) and with the tip (b), showing the field enhancement in the in-plane electric field caused by the tip.

To calculate the number of molecules involved in the strong coupling signature that is scattered by the tip and detected, the effective mode volume was extracted from the simulation, which has a field amplitude larger $\mathbf{1/e}$, to therefore account for the molecules experiencing the strongest field. This extracted volume was then matched with the thickness of the molecular layer and the overlapping volume was taken into account ($\approx \mathbf{4.5 * 10^4\ nm^3}$), leading to $\mathbf{3.8 * 10^4}$ molecules to be considered in the detected strong coupling phenomena.

5. CoPc thin film

Extraction of CoPc permittivity

To extract the permittivity of CoPc, a 650 nm thick layer was deposited on a CaF_2 substrate, and the transmission spectrum was measured with FTIR. Using[13]

$$\frac{T}{T_0} = \frac{1}{|1+\sigma(\omega)d\frac{Z_0}{n+1}|^2} \tag{S21}$$

With $\boldsymbol{d}$ being the layer thickness, $\boldsymbol{n}$ the refractive index of the substrate ($\boldsymbol{n \approx 1.37}$), $\boldsymbol{\sigma(\omega)}$ the complex conductivity of the CoPc film and $\boldsymbol{Z_0}$ the impedance of free space ($\boldsymbol{Z_0 = 377\Omega}$), the relationship between the complex conductivity $\boldsymbol{\sigma(\omega)}$ and the permittivity $\boldsymbol{\varepsilon(\omega)}$ is

$$\varepsilon(\omega) = 1 + \frac{i}{\omega\varepsilon_0}\sigma(\omega) \tag{S22}$$

We model the permittivity of CoPc in the region of interest with 4 Lorentz oscillators which represent the four molecular vibration dips.

$$\varepsilon(\omega) = \varepsilon_\infty + \sum_k \frac{S_k^2}{\omega_k^2 - \omega^2 - i\omega\gamma_k} \quad \textbf{(S23)}$$

Here, ε_∞ is the high-frequency dielectric constant, S_k the intensity, ω_k the central frequency and γ_k the damping of the oscillator.[12,14] The fit (red) and the experimental data (black) are shown in figure S16a. The corresponding fitting parameters can be found in table S2. Figure S16b shows the real (ε_1) and imaginary (ε_2) part of the dielectric permittivity extracted from the fit. ε_1 at 1122 cm^{-1} was found to be **2.7**.

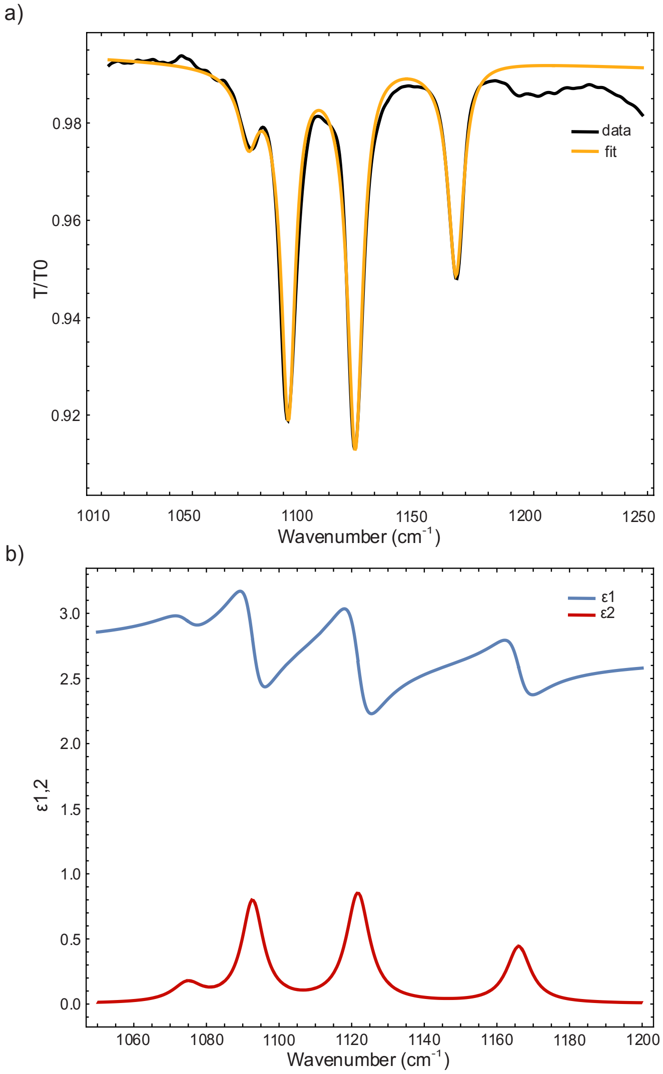

Figure S16. Extraction of the permittivity of Cobalt phthalocyanine (CoPc). (a) the transmission spectrum of 650 nm of CoPc on CaF_2 (black) and the fit (orange) to equation S23 (b) real ($\varepsilon_1 - \boldsymbol{blue}$) and imaginary ($\varepsilon_2 - \boldsymbol{red}$) part of the dielectric permittivity.

k	$\omega_k(cm^{-1})$	$\gamma_k(cm^{-1})$	$S_k(cm^{-1})$
1	1074.8	9.7	38.6
2	1092.7	7.2	78.1
3	1121.7	7.8	85.7
4	1166	8	63.8

Table S2. Figure S16 fitting parameters, $\boldsymbol{\varepsilon_\infty = 2.7}$.

Cobalt phthalocyanine thin film measured with OPTIR

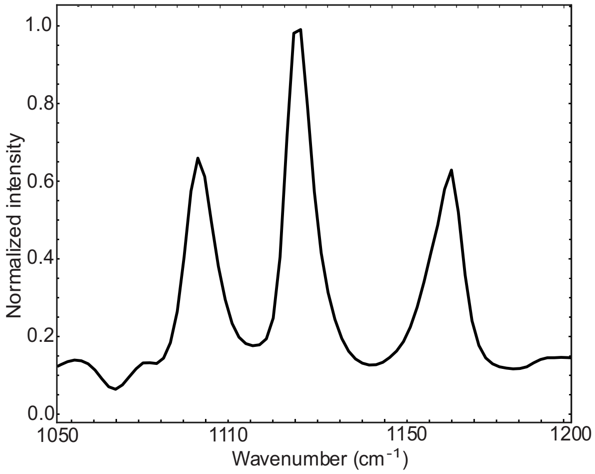

Figure S17. A thin layer of Cobalt phthalocyanine measured with optical photothermal infrared spectroscopy (OPTIR).

Cobalt phthalocyanine thin film measured with s-SNOM

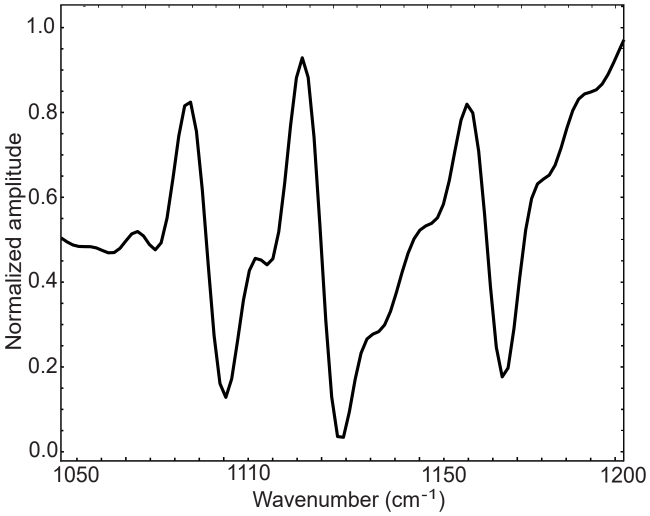

Figure S18. A thin layer of Cobalt phthalocyanine measured with s-SNOM, shown the 3rd harmonic amplitude.

The CoPc spectra obtained with OPTIR and s-SNOM do match well with each other in terms of frequency and relative intensity. It has to be noted that for a weakly coupled oscillator in s-SNOM a derivative line shape is obtained for the amplitude as the amplitude of the spectrum becomes approximately the real part of the dielectric function ϵ of the weak oscillator. This can account for the difference between the spectra taken by OPTIR (figure S17) and s-SNOM (figure S18). Further, this is not the case for a strongly coupled oscillator such as the strong coupling measured in this work as it is more close to absorption.[15]

6. s-SNOM

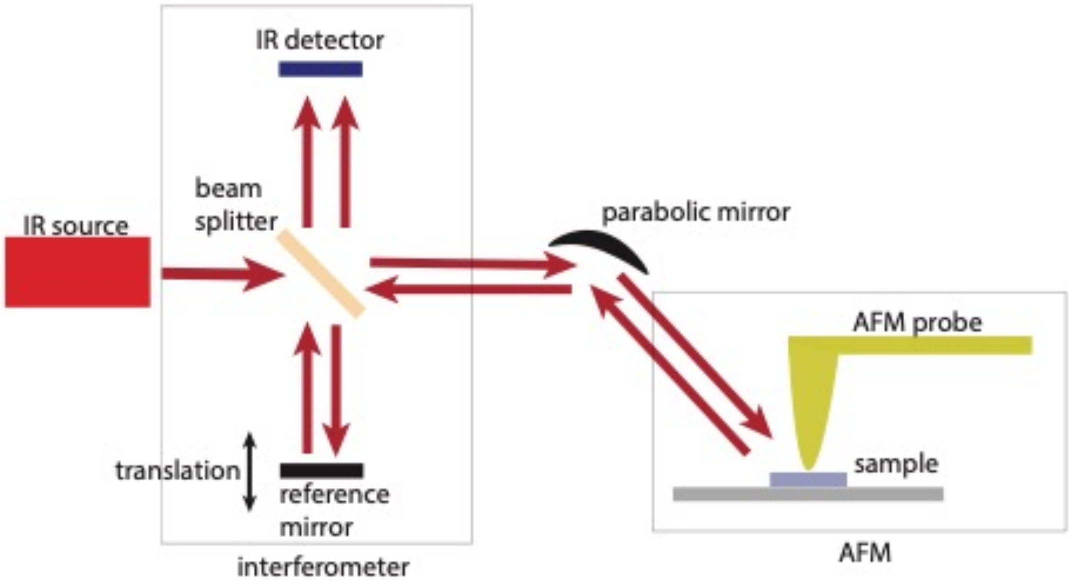

Figure S19. A schematic representation of a s-SNOM setup with a broadband infrared laser source.

s-SNOM, or scattering-Scanning Near-field Optical Microscopy, is a cutting-edge technique that enables high-resolution infrared spectroscopic characterization of materials at the nanoscale, well beyond the diffraction limit of traditional infrared microscopy. The measurement system utilized for this research is a commercially available system from Neaspec/Attocube.

In s-SNOM, an infrared laser source is integrated into an atomic force microscope (AFM) system. The laser beam is focused with a parabolic mirror (NA of ~0.5 and a solid angle of illumination of ~30°, focused beam size 10-15 μm) onto the apex of a

metallic coated AFM probe whose nanoscale apex is used as a near-field probe of the surface by acting as a localized scattering center of optical near-fields. The AFM probe tip is scanned above the sample surface using tapping mode feedback, typically with a probe oscillation amplitude of a few 10s of nanometers allowing for the near-field interaction between the tip and the sample surface.

As the AFM tip approaches the sample surface with each oscillation of the probe, the near-field interaction between the probe tip and the sample's surface modifies the local electromagnetic field distribution.[16,17] This interaction gives rise to a near-field signal detected via increased scattering of the localized probe tip, which contains valuable information about the sample's infrared absorption, reflection, or emission properties.

To acquire the s-SNOM spectra, an interferometric detection is employed. The near-field scattering signal from the AFM probe is combined with a reference signal, which is obtained by reflecting the laser light off a known reflector (reference mirror). The interference between the two signals is detected with a highly sensitive interferometer, enabling the extraction of the sample's infrared spectra with exceptionally high spatial resolution.[18,19]

As the AFM operates at the tapping mode, with a tapping frequency of ~285 kHz, the increased scattering associated with near-field coupling between the probe and surface can be detected by demodulating the scattering signal at the higher-harmonics of the tapping frequency. Consequently, the IR scattered signal was modulated at these higher frequencies after being picked up by the detector. We analyzed the 3rd harmonic of the modulated signal to more effectively remove the background from the spectrum. The interferometer center position is 400 µm and scanned for ± 400 µm, with 256 pixels / spectral bandwidth and 10.2 ms integration time per pixel to ensure a spectral resolution of 6.25 cm^{-1}, it was averaged for 30 times. It takes 1.5 min to acquire a single IR spectrum.

7. Pillar resonator and CoPc coupling fitting parameters

The fitting method for the fitted spectra shown in this section can be found in section 3, harmonic oscillator fitting model. The fitting was done with equation S10. We have investigated the influence of the additional modes (Table S2) but found their influence to be neglectable.

Fittings were done with both $F_m = 0$ and $F_m \neq 0$ for comparison, shown in figure S20. It can be noted that no significant improvement was found with $F_m \neq 0$, therefore the final fits were done with $F_m = 0$.

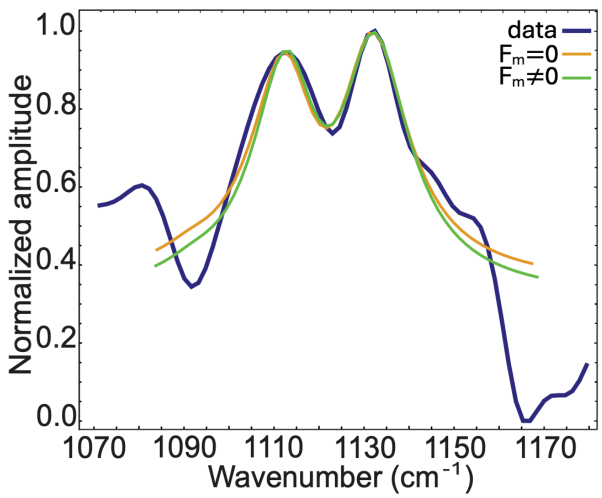

Figure S20 Fitted experimental s-SNOM data – comparison between $F_m = 0$ and $F_m \neq 0$.

	ω_p (cm-1)	ω_m (cm-1)	γ_p (cm-1)	γ_m (cm-1)	g (cm-1)	F_p	F_m	ϕ	$(\gamma_p + \gamma_m)/4$ (cm-1)
$F_m = 0$	1123	1122	22	13	9.7	4.5	0	0	8.75
$F_m \neq 0$	1123.2	1122	22	13	9.7	4.3	0.21	0	8.75

Table S3. Figure S20 fitting parameters

The spectrum of Figure 1 obtained at the edge of the quartz resonator covered with 20 nm of Cobalt phthalocyanine was fitted with equation (4) and plotted in figure 1b. The corresponding fitting parameters are shown in Table S4.

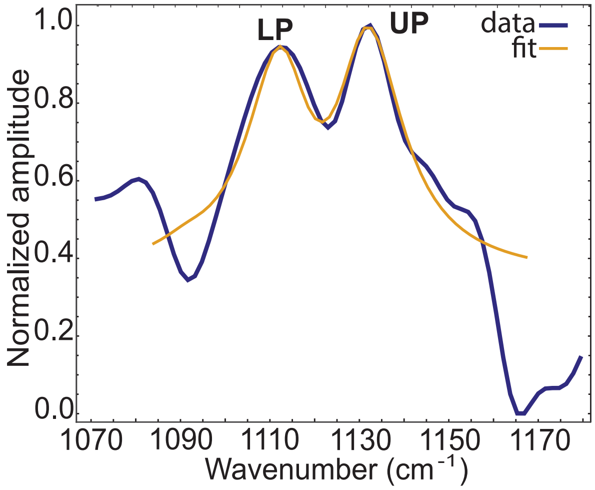

Figure S21. Fitted experimental s-SNOM data – Figure 1b.

Pillar diameter (µm)	ω_p (cm-1)	ω_m (cm-1)	γ_p (cm-1)	γ_m (cm-1)	g (cm-1)	F_p	ϕ	$(\gamma_p + \gamma_m)/4$ (cm-1)
1.6	1123	1122	22	13	9.7	4.5	0	8.75

Table S4. Figure 1b/S21 fitting parameters

In figure 2 and 3 of the main text, position 3 showed the maximum intensity. Position 3 was normalized, and all the other positions were divided by the same number respectively. Hence, positions 1-2 and 4-6 are normalized with respect to figure 3. This was done for figure 2 and 3 individually.

The figures and tables below are the detailed data analysis summarized in Figure 4 of the main text.

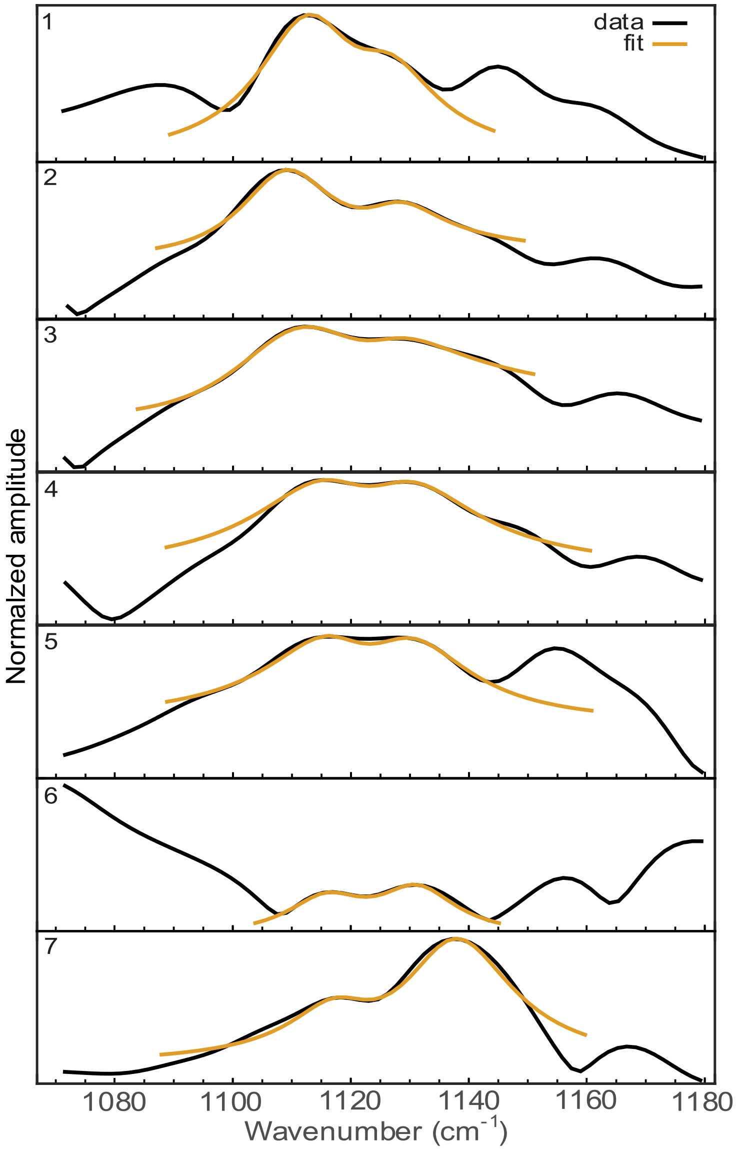

Figure S22. Fitted experimental s-SNOM data – 8 nm CoPc.

8nm	Pillar diameter (μm)	ω_p (cm-1)	ω_m (cm-1)	γ_p (cm-1)	γ_m (cm-1)	g (cm-1)	F_p	ϕ	$(\gamma_p+\gamma_m)/4$ (cm-1)
1	2.5	1111	1122	23	15	6	4.7	0.0	9.5
2	2.2	1114	1122.2	22	14	8.3	4.0	-0.2	9
3	1.9	1115	1122.1	35	16	6.5	5.0	0.0	12.75
4	1.6	1119	1121.9	35	16	7	5.0	-0.3	12.75
5	1.4	1122	1121.9	30	15	6.3	4.0	0.0	11.25
6	1.2	1124	1122	17	15	7.5	3.0	0.0	8
7	1.0	1132	1122.1	25	15	8.2	5.0	0.0	10

Table S5. 8nm CoPc fitting parameters – Figure S22.

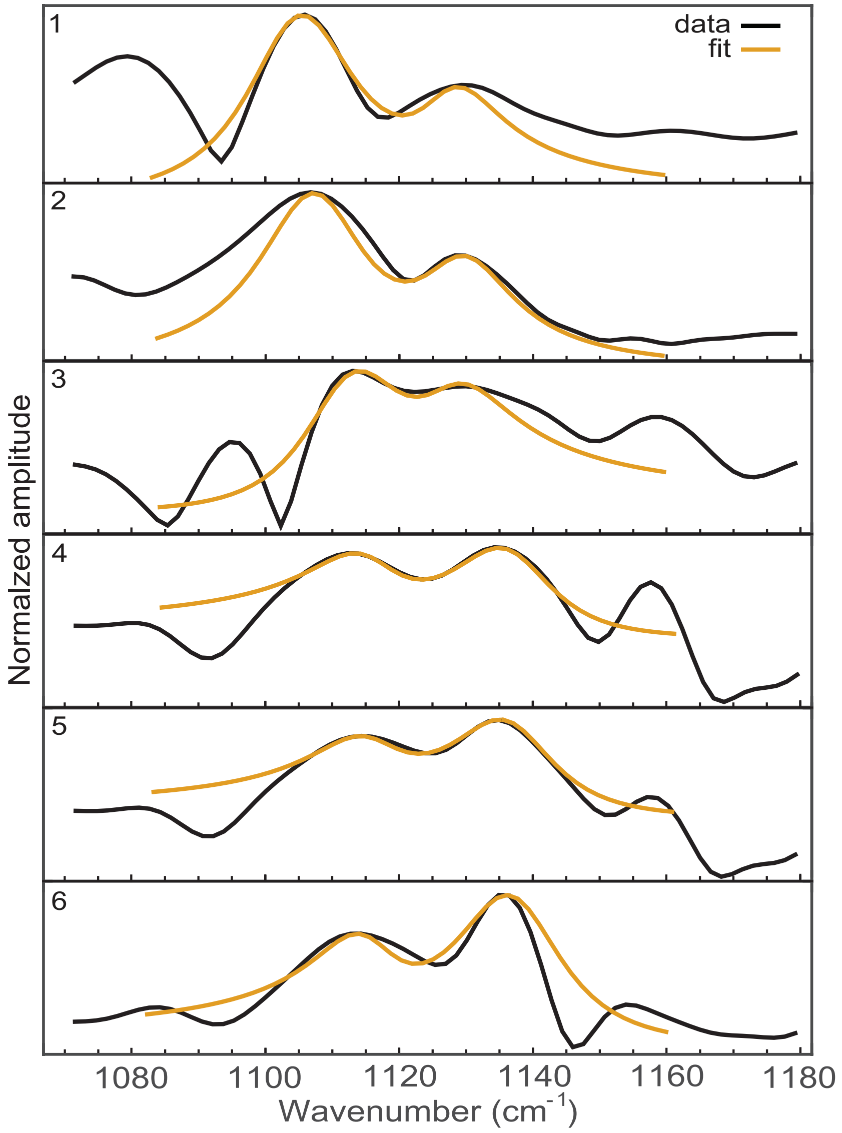

Figure S23. Fitted experimental s-SNOM data – 20 nm CoPc.

20nm	Pillar diameter (µm)	ω_p (cm-1)	ω_m (cm-1)	γ_p (cm-1)	γ_m (cm-1)	g (cm-1)	F_p	ϕ	$(\gamma_p + \gamma_m)/4$ (cm-1)
1	2.5	1110	1122.1	23	13	10	5.5	-0.2	9
2	2.2	1114.2	1122	23	15	10.2	5.5	0.0	9.5
3	1.9	1117	1121.9	23	15	8.5	5.0	-0.3	9.5
4	1.6	1124	1122	23	14	10.3	3.5	0.3	9.25
5	1.2	1128	1121.8	22	15	9.8	3.6	0.3	9.25
6	1.0	1131	1122.1	23	13	10	4.1	0.2	9

Table S6. 20nm CoPc fitting parameters – Figure S23.

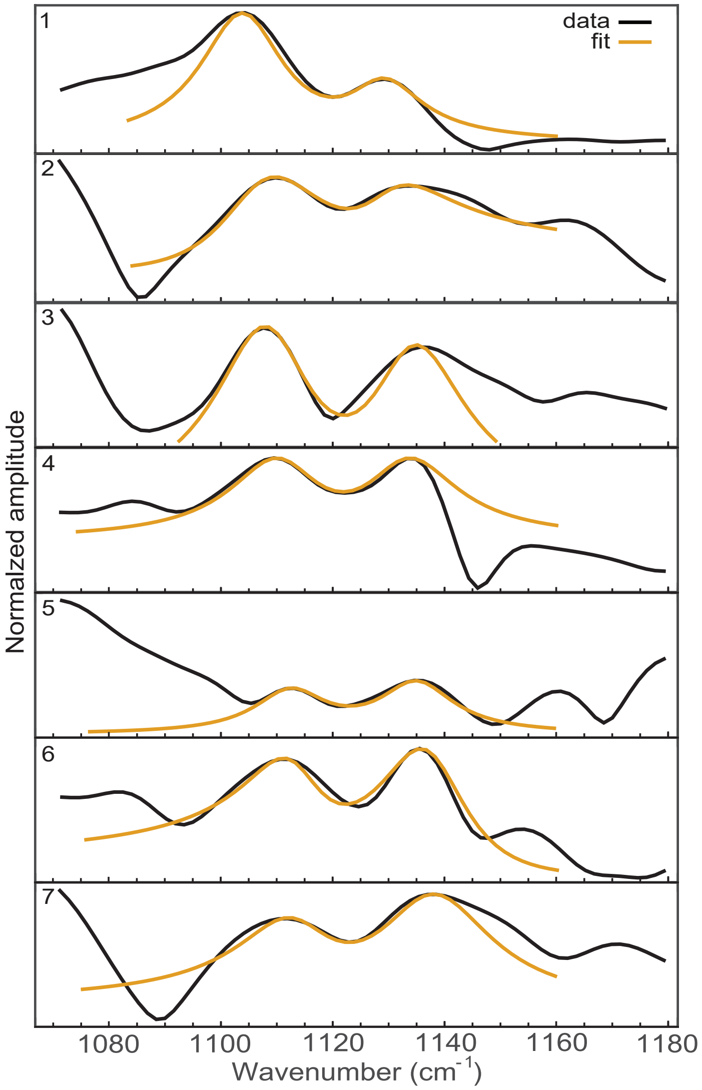

Figure S24. Fitted experimental s-SNOM data – 30 nm CoPc.

30nm	Pillar diameter (µm)	ω_p (cm⁻¹)	ω_m (cm⁻¹)	γ_p (cm⁻¹)	γ_m (cm⁻¹)	g (cm⁻¹)	F_p	ϕ	$(\gamma_p+\gamma_m)/4$ (cm⁻¹)
1	2.5	1110	1122	20	14	11	4.8	0.0	8.5
2	2.2	1113.5	1122.1	28	15	11	3.9	-0.3	10.75
3	1.9	1119.5	1121.9	27	15	13	6.3	0.0	10.5
4	1.6	1122	1122	25	14	11.3	4.0	0.0	9.75
5	1.4	1124.2	1122	17	15	11.3	3.2	0.0	8
6	1.2	1128	1122	14	14	11.8	4.5	0.32	7
7	1.0	1129	1122.1	30	16	12	5.0	0.11	11.5

Table S7. 30nm CoPc fitting parameters – Figure S24.

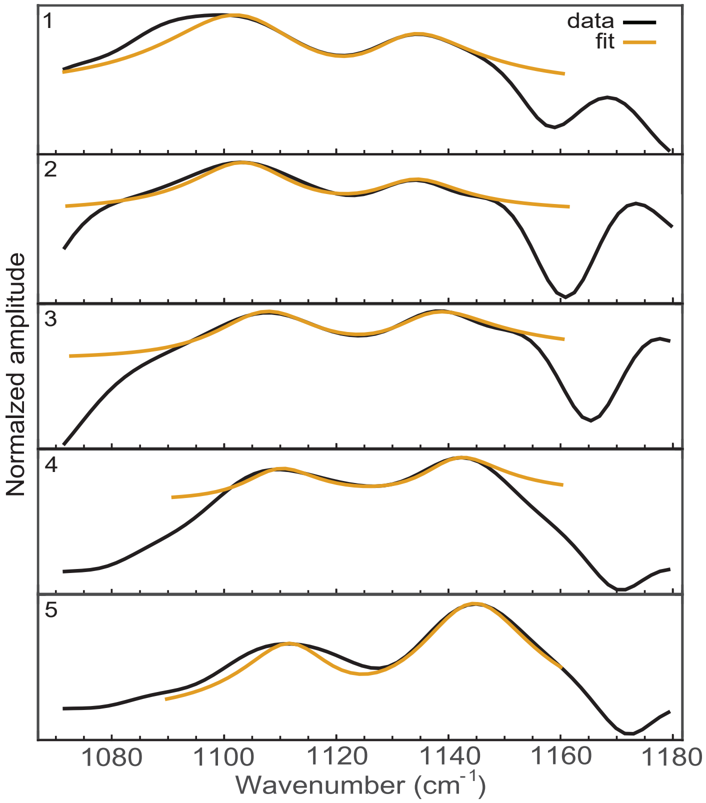

Figure S25. Fitted experimental s-SNOM data – 50 nm CoPc.

50nm	Pillar diameter (µm)	ω_p (cm⁻¹)	ω_m (cm⁻¹)	γ_p (cm⁻¹)	γ_m (cm⁻¹)	g (cm⁻¹)	F_p	ϕ	$(\gamma_p+\gamma_m)/4$ (cm⁻¹)
1	2.5	1112	1122	40	15	13.7	4.5	0.0	13.75
2	1.9	1115	1122.1	27	15	14	2.2	0.0	10.5
3	1.6	1119	1122	30	16	14.6	2.7	-0.3	11.5
4	1.2	1126	1121.8	20	15	15	2.7	-0.3	8.75
5	1.0	1132	1122.3	30	16	14.5	5.0	0.0	11.5

Table S8. 50nm CoPc fitting parameters – Figure S25.

8. References

(1) Bertolotti, M.; Sibilia, C.; M. Guzman, A. *Evanescent Waves in Optics*; Springer International Publishing: Cham, 2017; Vol. 206. https://doi.org/10.1007/978-3-319-61261-4.

(2) Jiang, K.; Lee, C.-H.; Jin, P. An Ultrathick SU-8 UV Lithographic Process and Sidewall Characterization. In *4M 2006 - Second International Conference on Multi-Material Micro Manufacture*; Elsevier, 2006; pp 211–216. https://doi.org/10.1016/B978-008045263-0/50049-0.

(3) Dolado, I.; Maciel-Escudero, C.; Nikulina, E.; Modin, E.; Calavalle, F.; Chen, S.; Bylinkin, A.; Alfaro-Mozaz, F. J.; Li, J.; Edgar, J. H.; Casanova, F.; Vélez, S.; Hueso, L. E.; Esteban, R.; Aizpurua, J.; Hillenbrand, R. Remote Near-Field Spectroscopy of Vibrational Strong Coupling between Organic Molecules and Phononic Nanoresonators. *Nat Commun* **2022**, *13* (1), 6850. https://doi.org/10.1038/s41467-022-34393-4.

(4) Wu, X.; Gray, S. K.; Pelton, M. Quantum-Dot-Induced Transparency in a Nanoscale Plasmonic Resonator. *Opt Express* **2010**, *18* (23), 23633. https://doi.org/10.1364/OE.18.023633.

(5) Zeb, M. A. Analytical Solution of the Disordered Tavis-Cummings Model and Its Fano Resonances. *Phys Rev A (Coll Park)* **2022**, *106* (6), 063720. https://doi.org/10.1103/PhysRevA.106.063720.

(6) Scully, M. O.; Zubairy, M. S. *Quantum Optics*; Cambridge University Press, 1997. https://doi.org/10.1017/CBO9780511813993.

(7) Chang, S.-W.; Chuang, S. L. Fundamental Formulation for Plasmonic Nanolasers. *IEEE J Quantum Electron* **2009**, *45* (8), 1014–1023. https://doi.org/10.1109/JQE.2009.2017210.

(8) Chang, S.-W.; Chuang, S. L. Normal Modes for Plasmonic Nanolasers with Dispersive and Inhomogeneous Media. *Opt Lett* **2009**, *34* (1), 91. https://doi.org/10.1364/OL.34.000091.

(9) Ahn, W.; Vurgaftman, I.; Dunkelberger, A. D.; Owrutsky, J. C.; Simpkins, B. S. Vibrational Strong Coupling Controlled by Spatial Distribution of Molecules within the Optical Cavity. *ACS Photonics* **2018**, *5* (1), 158–166. https://doi.org/10.1021/acsphotonics.7b00583.

(10) Chang, S.-W.; Chuang, S. L. Fundamental Formulation for Plasmonic Nanolasers. *IEEE J Quantum Electron* **2009**, *45* (8), 1014–1023. https://doi.org/10.1109/JQE.2009.2017210.

(11) Sauvan, C.; Hugonin, J. P.; Maksymov, I. S.; Lalanne, P. Theory of the Spontaneous Optical Emission of Nanosize Photonic and Plasmon Resonators. *Phys Rev Lett* **2013**, *110* (23), 237401. https://doi.org/10.1103/PhysRevLett.110.237401.

(12) Liu, K.; Huang, G.; Li, X.; Zhu, G.; Du, W.; Wang, T. Vibrational Strong Coupling between Surface Phonon Polaritons and Organic Molecules via Single Quartz Micropillars. *Advanced Materials* **2022**, *34* (8). https://doi.org/10.1002/adma.202109088.

(13) Glover, R. E.; Tinkham, M. Conductivity of Superconducting Films for Photon Energies between 0.3 and 40kTc. *Physical Review* **1957**, *108* (2), 243–256. https://doi.org/10.1103/PhysRev.108.243.

(14) Autore, M.; Li, P.; Dolado, I.; Alfaro-Mozaz, F. J.; Esteban, R.; Atxabal, A.; Casanova, F.; Hueso, L. E.; Alonso-González, P.; Aizpurua, J.; Nikitin, A. Y.; Vélez, S.; Hillenbrand, R. Boron Nitride Nanoresonators for Phonon-Enhanced Molecular Vibrational Spectroscopy at the Strong Coupling Limit. *Light Sci Appl* **2017**, *7* (4), 17172–17172. https://doi.org/10.1038/lsa.2017.172.

(15) Mester, L.; Govyadinov, A. A.; Hillenbrand, R. High-Fidelity Nano-FTIR Spectroscopy by on-Pixel Normalization of Signal Harmonics. *Nanophotonics* **2022**, *11* (2), 377–390. https://doi.org/10.1515/nanoph-2021-0565.

(16) Novotny, L.; Hecht, B. *Principles of Nano-Optics*; Cambridge University Press, 2012. https://doi.org/10.1017/CBO9780511794193.

(17) Keilmann, F.; Hillenbrand, R. Near-Field Microscopy by Elastic Light Scattering from a Tip. *Philosophical Transactions of the Royal Society of London. Series A: Mathematical, Physical and Engineering Sciences* **2004**, *362* (1817), 787–805. https://doi.org/10.1098/rsta.2003.1347.

(18) Amarie, S.; Ganz, T.; Keilmann, F. Mid-Infrared near-Field Spectroscopy. *Opt Express* **2009**, *17* (24), 21794. https://doi.org/10.1364/OE.17.021794.

(19) Huth, F.; Govyadinov, A.; Amarie, S.; Nuansing, W.; Keilmann, F.; Hillenbrand, R. Nano-FTIR Absorption Spectroscopy of Molecular Fingerprints at 20 Nm Spatial Resolution. *Nano Lett* **2012**, *12* (8), 3973–3978. https://doi.org/10.1021/nl301159v.
